# The underlying neurobiology of social isolation and loneliness in middle‐older adults: A systematic review

**DOI:** 10.1002/alz.71921

**Published:** 2026-10-28

**Authors:** Jade Guarnera, Katherine Kenyon, Neel Joshi, Karen Caeyenberghs, Michael Wheeler, Helen Macpherson

**Affiliations:** ^1^ Institute for Physical Activity and Nutrition Deakin University Geelong Victoria Australia; ^2^ Cognitive Neuroscience Unit, School of Psychology Deakin University Geelong Victoria Australia

**Keywords:** aging, brain aging, dementia risk, loneliness, neural correlates, neurobiology, neurodegeneration, neuroimaging, older adults, social health, social isolation

## Abstract

Social isolation and loneliness are distinct but overlapping dementia risk factors, though their underlying neurobiological mechanisms remain unclear. A systematic search identified 42 studies examining social isolation, loneliness, and neurobiological outcomes in middle‐aged and older adults, including structural and functional neuroimaging, neuroinflammatory, and neurodegenerative markers. Findings identified patterns of neurobiological associations that were reported exclusively for social isolation, exclusively for loneliness, and shared across both concepts. Social isolation was more frequently associated with temporal regions (middle temporal gyrus, temporal lobe), whereas loneliness demonstrated associations with subcortical structures (putamen, globus pallidus). Shared patterns of association were most evident in limbic regions (hippocampus, amygdala, thalamus, fusiform gyrus). Both constructs were also associated with greater white matter hyperintensity/lesion volume and elevated C‐reactive protein. Several implicated regions are vulnerable in dementia, suggesting potential neurobiological pathways linking poor social health to neurodegenerative risk. However, substantial methodological heterogeneity and inconsistent measurement approaches limit conclusions and highlight important directions for future research.

## Highlights

- This is the first systematic review to synthesize the neurobiological correlates of social isolation and loneliness in middle–older adults.
- Social isolation showed more consistent patterns of association with temporal brain regions.
- Loneliness showed more consistent patterns of association with subcortical structures.
- Shared neurobiological associations were most evident in limbic regions.
- Substantial methodological heterogeneity limited direct comparisons across studies.

## 1 INTRODUCTION

Older adults (typically defined at 60–65+ years)^1^, ^2^ are at heightened risk of experiencing loneliness and being socially isolated due to typical life changes associated with aging which can lead to being less socially engaged (e.g., death of spouse/friends, retirement, empty nests).^3^, ^4^, ^5^ Aside from this increased vulnerability, there is a continuing increase in the aging population;^6^, ^7^ hence, investigating the impact of social health on other health outcomes in older adultsis imperative.

While social isolation and loneliness are related, they fundamentally differ in their experiences and hence are distinct social health concepts.^5^, ^8^ Loneliness involves *subjective* negative feelings when there is a perceived discrepancy between one's actual and ideal quality/quantity of social interactions,^9^ while social isolation is an *objective* measure of the frequency of social interactions or the quantity of social connections within one's social network.^10^ The understanding of social isolation and loneliness has evolved; once used as interchangeable terms they are now recognized as distinct concepts that depict different social experiences.^8^ Both social health concepts are risk factors for numerous negative health outcomes (e.g., dementia, cognitive impairment, stroke, acute myocardial infarction). However, we currently lack a strong understanding of the mechanisms that might explain this risk, particularly the underlying neurobiology.

Social isolation and loneliness have been associated with distinct patterns in health risks.^11^, ^12^, ^13^ For example, a prospective study reported social isolation was more strongly associated with physical health outcomes (e.g., mortality risk), and loneliness with psychological outcomes (e.g., depression, life satisfaction, mental health).^13^ Poor social health has also been linked to dementia risk,^8^, ^14^ with one study reporting social isolation increasing the risk of dementia by 50%,^15^ and another study uncovering feelings of loneliness increased the risk of dementia by 31%.^16^

To further understand the neurodegenerative risk of poor social health, studies have investigated the underlying neurobiological mechanisms underpinning loneliness and social isolation. A range of neuroimaging scans and blood biomarkers have been used to examine the potential structural, functional, and neuroinflammatory substrates of heightened loneliness and social isolation. The social brain theoretical framework^17^ outlines key neural systems (e.g., white and gray matter, functional networks) involved in social cognitive processing, underpinning social functions such as mentalizing, empathy, and theory of mind (e.g., Wang and Olson^18^). However, this framework is not universally accepted, and its application to social isolation and loneliness research remains limited. Notably, much of the existing literature exploring the neurobiological associations of social isolation and loneliness has been conducted in younger adult samples (< 40 years; e.g., Cacioppo et al.,^19^ Brilliant et al.,^20^ and Hu et al.^21^), or animal studies inducing isolation (e.g., Tamano et al.,^22^ Corsi‐Zuelli et al.,^23^ and Niu et al.^24^). Given the increased vulnerability to social isolation, loneliness, and dementia onset in older adulthood, and the limited capacity of animal models replicating the distinct human social experience, research on the older adult population is imperative to understanding the neurobiological mechanisms of these social concepts.

To date, there are currently three systematic reviews that have synthesized the literature on this topic, with numerous of the included studies examining younger adult samples.^9^, ^25^, ^26^ However, Lam et al.’s review^9^ focused only on loneliness, Xiong et al.^26^ only explored social isolation, and Zovetti et al.^25^ included loneliness studies when exploring the neural basis of social isolation. This is a major challenge within the social health discipline, as using the terms social isolation and loneliness synonymously, interchangeably using measures designed for one when investigating the other, and conflating results/findings has resulted in inaccurate depictions of the social concepts (e.g., Zovetti et al.,^25^ Franck et al.,^27^ Seyfzadeh et al.,^28^ Harrington et al.,^29^ and Dodge et al.^30^). Moreover, this has hindered our ability to identify and differentiate the unique neurobiological mechanisms and pathways through which each social concept may contribute to dementia risk.

This systematic review aims to synthesize the existing literature investigating the neurobiological correlates of social isolation and loneliness in the middle–older adult population. Specifically, this review seeks to identify exclusive and shared patterns of association across gray and white matter characteristics, functional connectivity and neural activity, as well as neuroinflammatory and neurodegenerative markers. Within this framework, *exclusive patterns* refer to neurobiological associations reported for either social isolation or loneliness, but not both, whereas *shared patterns* refer to neurobiological associations reported for both constructs. As few studies examined both social isolation and loneliness within the same analytical framework, these classifications are based on patterns across studies rather than direct comparisons and should therefore be interpreted as indicative of broader trends rather than definitive neurobiological specificity. Furthermore, to support accurate interpretation, the review focuses on studies using accurate measures and definitions of social isolation and loneliness, to ensure these constructs are correctly represented within the literature.

## 2 METHOD

### 2.1 Search strategy

The current systematic review was conducted in accordance with the 2020 guidelines for the Preferred Reporting Items for Systematic Review and Meta‐Analysis (PRISMA‐2020)^31^ and was also registered on the PROSPERO database (ID: CRD420251029053). Papers were identified via an electronic search of PubMed, PsycInfo, Medline ProQuest, and Embase databases. The search syntax incorporated synonyms of terms related to neuroimaging and neurobiology measures, and outcomes related to brain structure/function/inflammation, including “magnetic resonance imaging,” “gray matter volume,” “neuroinflammation,” and “functional connectivity,” combined with various terms relating to our population of interest (e.g., “lonely,” “socially isolated”). The title/abstract search was limited to papers published within the past 15 years (2010–2025). A 15‐year inclusion window was selected as the understanding of social isolation and loneliness has evolved in recent years. Previously, these social concepts were used as interchangeable terms, while their distinction was recognized in the literature by 2010,^32^ and have since been increasingly considered distinct concepts that represent different social experiences.^8^ Furthermore, substantial advances in neuroimaging acquisition, processing, and analytical techniques have occurred over the past 15 years, significantly improving the quality and sensitivity of neuroimaging findings. Notably, the launch of the Human Connectome Project in 2010 was associated with major developments in multimodal neuroimaging acquisition, processing, and analysis.^33^ We therefore selected 2010 as a pragmatic boundary intended to improve the conceptual and methodological comparability of the included evidence while retaining a sufficiently broad evidence base. Additionally, only human studies were included, and age range was capped to middle–older adult samples. For our complete list of search terms, see Supplementary Materials in supporting information.

**RESEARCH IN CONTEXT**

1. **Systematic review**: The authors reviewed the literature using PubMed, PsycInfo, Medline ProQuest, and Embase databases in accordance with Preferred Reporting Items for Systematic Review and Meta‐Analysis guidelines. While social isolation and loneliness have increasingly been implicated as a dementia risk factors, their underlying neurobiological associations remain unclear. This review systematically synthesized evidence examining the neuroimaging, neuroinflammatory, and neurodegenerative correlates associated with social isolation and loneliness in middle‐aged and older adults, including patterns of association reported exclusively for each contrast and those shared across both.
2. **Interpretation**: Findings suggest that social isolation and loneliness demonstrate both exclusive and shared patterns of neurobiological associations. Shared associations were most commonly observed in limbic regions. Social isolation was more consistently associated with temporal regions, whereas loneliness was more consistently associated with subcortical structures.
3. **Future directions**: Future research should focus on directly comparing social isolation and loneliness within the same analytical frameworks, and address methodological heterogeneity issues. Longitudinal multimodal neuroimaging studies are needed to clarify whether these neurobiological patterns contribute to dementia‐related neurodegeneration and establish the specificity of neural correlates associated with social isolation and loneliness.

### 2.2 Eligibility criteria

Following the Population/Intervention/Comparator/Outcome (PICO) guidelines, papers were included at the title/abstract screening level if they measured either loneliness, social isolation, or both (measured during adulthood) along with a neurobiological measure (i.e., gray/white matter volume, functional connectivity, neuroinflammation; “Outcome”) and were published from 2010 to 2025. Only studies using measures that accurately operationalized social isolation and/or loneliness, in accordance with their respective definitions, were eligible for inclusion. If a study examined both constructs but only one was measured accurately, only the findings relating to the correctly operationalized construct were included in the structured narrative synthesis. We restricted our search to studies with a mean sample age of ≥ 50 years to capture adults in late midlife and older adulthood, the period during which neurobiological changes associated with dementia risk typically begin to emerge, often preceding the onset of clinical symptoms (“Population,” “Intervention”).^34^, ^35^ Additionally, preliminary searches indicated that relatively few neurobiology studies were conducted exclusively in older adult (≥ 60–65 years) populations. Therefore, including adults within late midlife enabled a more comprehensive synthesis of available evidence, moreover maintaining a focus on populations relevant to dementia risk. Papers were included irrespective of whether they included a non‐lonely/non‐isolated control group (“Comparator”). Studies were included if they conducted at least one analysis examining associations among loneliness, social isolation, and neurobiological measures, irrespective of additional cognitive or behavioral outcomes.

Participants with cognitive impairment, dementia (including Alzheimer's disease), or depression (including major depressive disorder) were eligible for inclusion. We excluded participants with other clinical diagnoses or conditions. These eligibility criteria were selected to capture evidence across the cognitive‐health continuum relevant to dementia, including cognitively unimpaired populations, cognitive impairment, and dementia. Depression was also included due to its close association and symptomatic overlap with loneliness, as well as its potential relevance to dementia risk.^36^, ^37^ Other clinical conditions were excluded due to their potential independent influences on neurobiological outcomes and the introduction of heterogeneity beyond the scope of this review. For studies that included both cognitively healthy participants and participants with cognitive impairment, dementia, or depression, results were eligible only if they were analyzed and reported separately for each group. Analyses that pooled clinical and cognitively healthy participants were excluded. Furthermore, the following were excluded, animal studies, samples with < 10 human participants and case studies, non‐primary research (i.e., reviews, meta‐analyses), full‐text or English transcribed papers that were unavailable.

Although one of the aims of this review is to discuss quality studies that included strong forms of measures and accurate definitions of loneliness and social isolation, papers which used single‐item measures were still included for a critical discussion of their findings.

### 2.3 Study selection

A PRISMA flowchart of all stages of the study selection process is shown in Figure 1. The search of the aforementioned databases was conducted on April 11, 2025, which yielded 185 records, and an additional search was conducted on April 14, 2026 to capture any newly published studies, identifying a further five records. After peer review, an additional Embase search was conducted on August 4, 2026, identifying three further records. Records identified across all database searches were combined, and 40 duplicates were removed using Zotero's deduplication tool. Title and abstract screening of 162 records was conducted independently using Rayyan^38^ by researchers J.G. and K.K. according to the predefined eligibility criteria described in section 2.2. When eligibility could not be determined by the title/abstract, papers were retained for full‐text assessment. Screening resulted in 70 papers excluded due to not meeting the eligibility criteria. Full‐text assessment of the remaining 92 reports by J.G. and K.K. led to a further 50 reports being excluded, resulting in 42 included studies. Discrepancies at each screening stage were discussed with the researchers involved with screening and consensus was reached for all final decisions.

**FIGURE 1:**
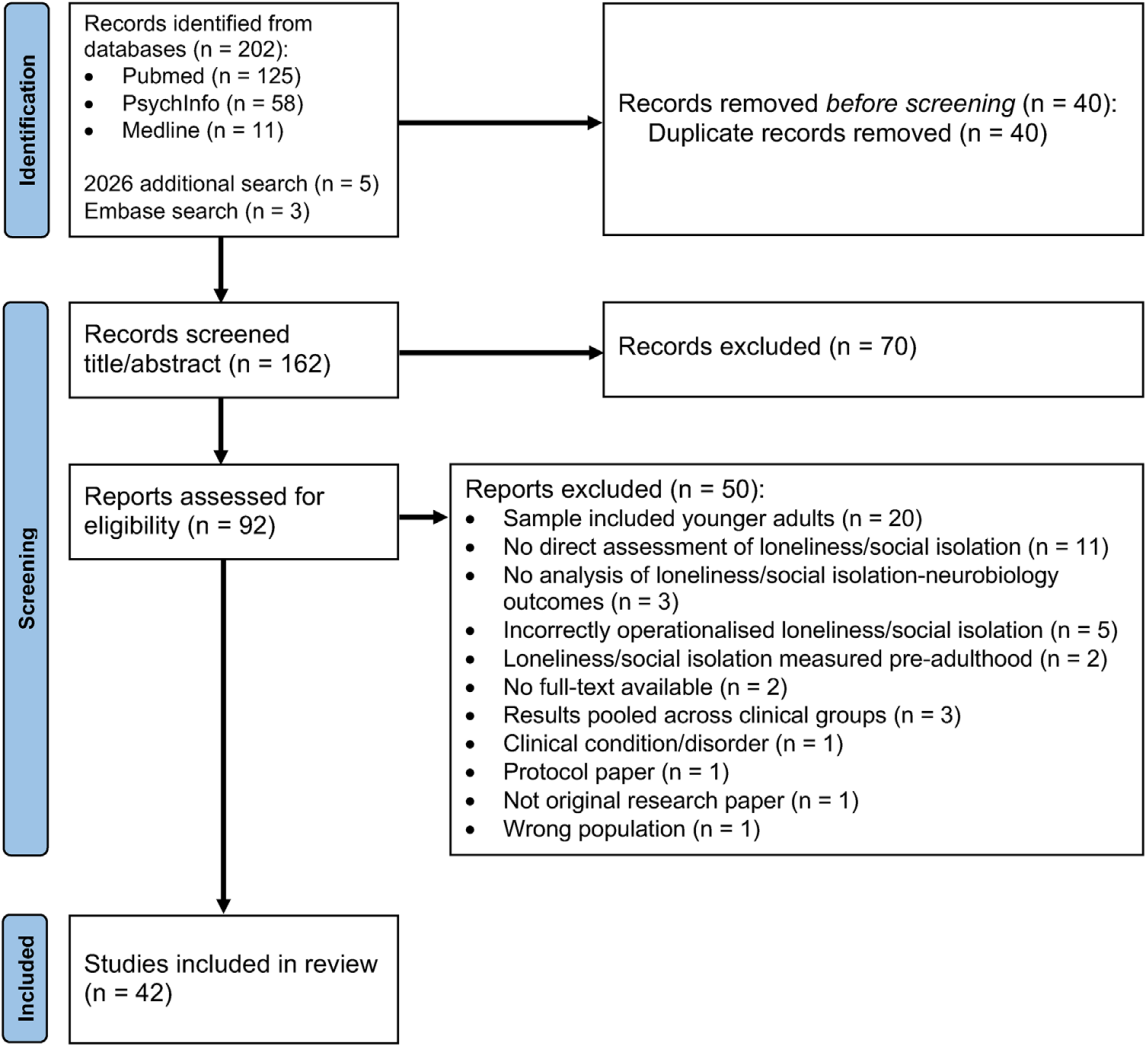
Preferred Reporting Items for Systematic Review and Meta‐Analysis flowchart.

### 2.4 Data extraction

Data extraction from all eligible studies for qualitative synthesis was performed by one reviewer (J.G.). Specifically, the following information was extracted: study details (author, year), study design, sample characteristics (size, age, sex), tool(s) used to measure social isolation and loneliness (e.g., University of California Los Angeles Loneliness Scale [UCLA‐LS], living alone), type of neurobiological outcome (e.g., gray matter volume, C‐reactive protein concentration), neuroimaging technique (e.g., magnetic resonance imaging [MRI], functional MRI [fMRI], positron emission tomography [PET]), and key findings (statistically significant and non‐significant).

### 2.5 Data synthesis

This review used a structured narrative synthesis approach, as a meta‐analysis could not be undertaken due to substantial heterogeneity involving measures used to assess social isolation and loneliness, the neurobiological outcomes examined, and statistical reporting across studies. To assess the feasibility of a quantitative synthesis, the included studies were examined for reporting quantitative measures of association (i.e., correlation coefficients, standardized regression coefficients, effect size). However, these measures were inconsistently reported, with numerous studies either not reporting or providing insufficient information to derive effect estimates.

Within this structured narrative synthesis, findings were first grouped according to the social concept examined: social isolation (Table 1), loneliness (Table 2), and both (Table 3). For studies examining both social isolation and loneliness, findings were separated and grouped according to their respective social construct for synthesis and reporting within the Results. Findings were then grouped according to the neurobiological outcomes examined and measures used to assess them, including structural MRI (gray and white matter volume, white matter hyperintensities, total brain volume, intracranial volume, morphometry‐derived brain age, cortical thickness, total cranial volume), resting‐state and task‐based fMRI (functional activity, functional connectivity), diffusion tensor imaging (DTI; white matter tract integrity, structural connectivity), PET (tau, amyloid beta [Aβ]), cerebrospinal fluid (CSF; tau, Aβ, phosphorylated tau [p‐tau]), blood‐based neuroinflammatory markers (C‐reactive protein, albumin, fibrinogen), perfusion MRI/arterial spin labelling (cerebral blood flow). Both statistically significant and non‐significant results were extracted and tabulated for each study and considered within these groupings during the narrative synthesis. Neurobiological findings were then compared between social isolation and loneliness studies to identify patterns of association reported exclusively for each concept and shared patterns between the two. “Exclusive patterns” were defined as neurobiological findings that were significantly associated with either social isolation *or* loneliness but not both across the literature, with significant associations reported in at least two studies. “Shared patterns” were defined as neurobiological findings that were significantly associated with both constructs across the literature, with at least one significant association reported for each social construct.

**TABLE 1:** Studies that investigated social isolation only.

Author/title	Sample Group/*n* (Age mean [SD], range) *Female/male*	Study design	Social isolation measure	Neurobiology measures	Key findings (Green = significant, red = non‐significant)
**Flatt et al., 2015** Social Network Size and Findings on Cranial Magnetic Resonance Imaging in Older Adults: The Cardiovascular Health Study	**1580 older adults** (79.3 [4.1]) *NA*	Cross‐sectional	Lubben Social Network Scale – social network categorical groups: very small, small, medium, large	WMHs (MRI)	Very small social networks significantly increased odds of WMHs compared to larger social networks. Smaller family network significantly associated with severe WMHs.
					No significant association between friendship network and WMHs.
**Groechel et al., 2024** Associations between mid‐life psychosocial measures and estimated late life amyloid burden: The Atherosclerosis Risk in Communities (ARIC)‐PET study	**316 middle‐aged adults** (55.1 [5.2], 45–65) *177 F/139 M*	Cross‐sectional	Lubben Social Network Scale – isolation categorical groups: isolated/high risk, moderate risk, low risk	Aβ (PET—florbetapir SUVRs)	Participants at low risk of social isolation were significantly more likely to have elevated global Aβ burden compared to moderate‐risk group.
					No significant findings for isolated/high‐risk group.
**Hirabayashi et al., 2023** Association between frequency of social contact and brain atrophy in community‐dwelling older people without dementia	**8896 older adults no dementia** (73.0 [6.0], > 65) *5111 F/378 5M*	Cross‐sectional	Frequency of contact with non‐cohabitating friends and relatives— isolation categorical groups: everyday, several times a week, several times a month, seldom.	GM volume, WMLV, TBV (MRI) ROI‐based analysis: frontal, parietal, temporal, occipital lobes, insula, cingulum, hippocampus, amygdala, thalamus, caudate, putamen, accumbens, globus pallidus	Low social contact (seldom/several times a month) was significantly associated with smaller volume within the temporal lobe, occipital lobe, cingulum, hippocampus, amygdala, and superior temporal gyrus. Seldom social contact was significantly associated with smaller volume within the inferior temporal gyrus, transverse temporal gyrus, entorhinal cortex, and rostal anterior cingulate gyrus. Several times a month social contact was significantly associated with reduced volume within the middle temporal gyrus and fusiform gyrus. Seldom social contact was significantly associated with lower total brain volume. Low social contact significantly associated with increased white matter lesion volume. Depressive symptoms accounted for 15%–29% of the associations between lower social contact and each regional volume.
**Hou et al., 2021** Associations of healthy lifestyles with cerebrospinal fluid biomarkers of Alzheimer's disease pathology in cognitively intact older adults: the CABLE study	**1108 cognitively intact middle–older adults** (61.1 [11.0], 40–90) *461 F/647 M*	Cross‐sectional	Social isolation: lives alone or no close relationship with relatives/friends/neighbors	Aβ, tau total, p‐tau (CSF)	Socially engaged participants exhibited significantly lower CSF tau and p‐tau compared to those who were socially isolated.
					No significant association with Aβ burden.
**Lammer et al., 2023** Impact of social isolation on grey matter structure and cognitive functions: A population‐based longitudinal neuroimaging study	**1409 middle–older adults (follow‐up)** (68.0 [7.0], 50–84) *656 F/753 M*	Longitudinal	Lubben Social Network Scale	GM volume, cortical thickness (MRI) Whole‐brain analysis	Higher baseline social isolation and increases in social isolation over a 6‐year period were significantly associated with smaller hippocampal volume. Social isolation at baseline was significantly associated 8 clusters displaying decreased cortical thickness (left precuneus, cuneus, precentral gyrus and posterior cingulate gyrus, and right supramarginal gyrus and cuneus). Increases in social isolation over time was significantly associated with decreased cortical thickness in one cluster (right superior frontal gyrus).
					No clusters from the previous models remained significant after controlling for cardiovascular covariates.
**Murayama et al., 2025** Impact of social isolation on change in brain volume in community‐dwelling older Japanese people: The NEIGE Study	**279 older adults** (73.0 [5.1], 65–84) *146 F/133 M*	Longitudinal	Frequency of social contact (poor social network) Living alone (household composition)	GM volume (MRI) ROI‐based analysis: hippocampus	Low‐frequency social contact was significantly associated with a decline in hippocampal volume over time, independent of loneliness.
					Living alone not significantly associated with significant changes in hippocampal volume. No significant association between frequency of social contact or household composition with changes in total GM volume.
**Qi et al., 2023** Sex differences in the mediating role of chronic inflammation on the association between social isolation and cognitive functioning among older adults in the United States	**2535 older adults** (71.05 [7.77], > 60) *1268 F/1267 M*	Cross‐sectional	Social Network Index	CRP, fibrinogen, albumin (blood samples)	Social isolation in older men was significantly associated with higher levels of inflammation measured by log‐transformed CRP and fibrinogen.
					Social isolation in older women was not significantly associated with log‐transformed CRP and fibrinogen. Socially isolated older women exhibited slightly elevated fibrinogen, not significant after adjusting for covariates (age, race/ethnicity, education, annual household income, smoking status, physical activity, obesity, hypertension, diabetes mellitus).

Abbreviations: Aβ, amyloid beta; CRP, C‐reactive protein; CSF, cerebrospinal fluid; GM, gray matter; HC, healthy control; MRI, magnetic resonance imaging; PET, positron emission tomography; p‐tau, phosphorylated‐tau; ROI, region of interest; SD, standard deviation; SUVR, standardized uptake value ratio; TBV, total brain volume; WMHs, white matter hyperintensities; WMLV, white matter lesion volume.

**TABLE 2:** Studies that investigated loneliness only.

Author/title	Sample Group/*n* (Age M [SD], range) *Female/male*	Study design	Loneliness measure	Neurobiology measures	Key findings (Green = significant, red = non‐significant)
**Cao et al., 2026** Effect of loneliness on social exclusion among older adults: A functional magnetic resonance imaging study	**40 cognitively healthy older adults** (61–69) *18 F/22 M* **21 lonely** (64.40 [2.04]) *7 F/14 M* **16 non‐lonely** (65.01 [2.72]) *9 F/7 M*	Cross‐sectional	De Jong‐Gierveld Loneliness Scale	Task‐evoked functional activity (fMRI task‐based) Task: Cyberball social exclusion task	Lonely older adults exhibited significantly greater functional activation during the social exclusion block within the bilateral medial superior frontal gyrus, bilateral medial orbital part of superior frontal gyrus, left anterior cingulate gyrus, left middle frontal gyrus, left superior dorsolateral frontal gyrus compared to the non‐lonely group. Within the non‐lonely group, older adults’ functional activation during the social inclusion block was greater than during the exclusion block, particularly within the medial prefrontal cortex, precuneus, inferior parietal/angular gyrus, and temporal cortices.
**Chen et al., 2022** Individual Differences in Cerebral Perfusion as a Function of Age and Loneliness	**45 middle–older adults** (63.4 [6.5], 53–81) *33 F/12 M* **44 young adults** (20.9 [3.2], 18–36) *36 F/8 M*	Matched cohort study	UCLA‐LS	Cerebral blood flow (MRI—ASL)	Middle‐older adults reporting high loneliness exhibited significantly greater cerebral blood flow within the bilateral superior temporal gyrus and left supramarginal gyrus compared to younger adults reporting high loneliness.
					No significant main effect of loneliness on cerebral blood flow in middle–older adults.
**d'Oleire Uquillas et al., 2018** Regional tau pathology and loneliness in cognitively normal older adults	**117 cognitively normal older adults** (76.01 [6.22], 65–92) *69 F/48 M*	Cross‐sectional	UCLA‐LS (3‐item)	Tau (PET—flortaucipir) ROIs: entorhinal, inferior temporal cortices	Greater tau pathology in right entorhinal cortex was significantly associated with greater loneliness, which remained significant after controlling for social network, depression, anxiety, memory, SES. Exploratory whole‐brain surface maps also identified an additional significant positive correlation between tau within the right fusiform gyrus and greater loneliness.
					No significant associations between tau pathology within inferior temporal cortex or left entorhinal with loneliness.
**Donovan et al., 2016** Association of higher cortical amyloid burden with loneliness in cognitively normal older adults	**79 cognitively normal older adults** (72.4 [6.2], 65–90) *43 F/36 M*	Cross‐sectional	UCLA‐LS (3‐item)	Aβ (PET—Pittsburgh Compound‐B)	Greater amyloid burden was significantly associated with greater loneliness. Amyloid‐positive participants were more likely to experience loneliness than amyloid negative.
**Düzel et al., 2019** Structural Brain correlates of Loneliness among older Adults	**319 older adults** (70.1 [3.7], 61–82) *122 F/197 M*	Cross‐sectional	UCLA‐LS	VBM cluster analysis of GM volume (MRI). Both whole‐brain and ROI‐based analyses: medial orbitofrontal cortex, dorsolateral prefrontal cortex, anterior cingulate cortex, insula, amygdala, hippocampus, nucleus accumbens	Greater loneliness significantly associated with reduced GM volume in 3 clusters: (1) amygdala, anterior hippocampus, adjacent entorhinal/parahippocampal cortex; (2) left posterior parahippocampal gyrus; (3) cerebellum, while controlling for social network size.
**Ehlers et al., 2017** Regional brain volumes moderate, but do not mediate, the effects of group‐based exercise training on reductions in loneliness in older adults	**247 older adults** (65.4 [4.6], 60–79) *169 F/78 M*	RCT	UCLA‐LS	GM volume (MRI) ROI‐based analysis: amygdala, hippocampus, PFC	Changes in loneliness after 6‐month RCT had no significant impact on amygdala, PFC, or hippocampal volume.
**Gao et al., 2020** The relationship between loneliness and working memory–related frontoparietal network connectivity in people with major depressive disorder	**21 medicated MDD patients middle‐aged adults** (52.0 [5.0]) *15 F/6 M* **24 HC** (48.7 [6.0]) *19 F/5 M*	Matched case–control	UCLA‐LS	FC (fMRI task‐based) Task: n‐back	Loneliness in HCs exhibited significantly stronger positive FC between the inferior parietal cortex and a rostral‐ventral part of the dorsomedial PFC compared to the MDD group. There was a significant interaction effect between MDD status and loneliness on FC between the supplementary motor area and caudal dorsomedial PFC during task performance, loneliness in HCs exerted a more positive effect with FC compared to in the MDD group. However, only marginally significant after false discovery rate correction.
**Kehrer‐Dunlap et al., 2022** Higher amyloid is associated with greater loneliness among cognitively normal older adults during the COVID‐19 pandemic	**108 cognitively normal older adults** (75.0 [5.5], > 65) *58 F/50 M*	Cross‐sectional	UCLA‐LS	Aβ (PET—Pittsburgh compound B, florbetapir)	Significant association between greater loneliness and higher amyloid accumulation; however, a weak association.
**Lan et al., 2015** Functional Connectivity Density Mapping of Depressive Symptoms and Loneliness in Non‐Demented Elderly Male	**85 older adults** (80.3 [5.6], > 65) *85 M*	Cross‐sectional	UCLA‐LS	FC (rsfMRI)	Significant positive association between loneliness and short‐range FC density within the bilateral lingual gyrus.
					No significant associations between loneliness and long‐range FC density.
**Lee et al., 2024** Association between loneliness and cognitive function, and brain volume in community‐dwelling elderly	**513 older adults** (71.0 [4.1], > 65) *304 F/209 M* **272 middle‐aged adults** (60.4 [2.7], 55–64) *189 F/83 M* Combined middle‐older adults in analyses.	Cross‐sectional	UCLA‐LS & CES‐D‐K (1‐item) Lonely subgroups based on each measure	GM + WM volume, TBV, WMH volume, cortical thickness (MRI, DTI) ROI‐based analysis: limbic system, lobar, basal ganglia	Loneliness measured via UCLA‐LS significantly associated with reduced volume in cerebral WM, bilateral frontal WM, left thalamus, left globus pallidus, and larger WMH volume. Loneliness measured via CES‐D‐K significantly associated with lower TBV, cerebral GM, subcortical GM, bilateral globus pallidus, putamen, left thalamus, right hippocampus, and larger WMH volume.
					Neither loneliness measure was significantly associated with whole‐brain cortical thickness.
**Park et al., 2023** Association between Loneliness and Memory Function through White Matter Hyperintensities in Older Adults: The Moderating Role of Gender	**173 older adults** (71.87 [6.79], 60–93) *103 F/71 M*	Cross‐sectional	UCLA‐LS‐Revised	WMHs (MRI)	Loneliness significantly positively correlated with WMHs, though this association was weak.
**Salinas et al., 2022** Association of Loneliness With 10‐Year Dementia Risk and Early Markers of Vulnerability for Neurocognitive Decline	**1611 MRI subsample middle–older adults** (62.0 [9.0], 40–79) *NA*	Retrospective	CES‐D (1‐item)	GM volume, TCV, WMHs (MRI) ROI‐based analysis: hippocampus	Lonely participants exhibited significantly lower TCV and greater WMH burden compared to non‐lonely.
					No significant association between loneliness and hippocampal volume.
**Saris et al., 2022** Social dysfunction is transdiagnostically associated with default mode network dysconnectivity in schizophrenia and Alzheimer's disease	**47 AD middle‐older adults** (68.4 [7.3]) *20 F/27 M* **27 HC** (66.8 [7.0], 50– 80) *13 F/14 M*	Cross‐sectional	De Jong‐Gierveld Loneliness Scale	FC (rsfMRI) ROIs: DMN	Loneliness in both AD and HC groups was significantly associated with diminished DMN FC within the rostromedial prefrontal sub‐territory of the DMN.
**Shao et al., 2020** Loneliness and depression dissociated on parietal‐centered networks in cognitive and resting states	**27 MDD middle‐aged adults** (51.85 [5.01], 39–59) *20 F/7 M* **25 HC** (48.72 [5.86]) *19 F/6 M*	Cross‐sectional	UCLA‐LS	FC (rsfMRI, task based) Task: Stroop color‐word	During the Stroop task, loneliness across all participants was significantly associated with increased parietal FC with the rostral anterior cingulate cortex, dorsal anterior cingulate cortex, left dorsolateral PFC, and right inferior and superior parietal cortices. Loneliness in MDD group significantly exhibited reduced FC in these regions, while HC loneliness was associated with stronger FC. During resting state, loneliness in HCs was significantly associated with stronger FC between the parietal cortex and anterior cingulate cortex, and between the cerebellum and anterior cingulate cortex, compared to the MDD group. Exploratory analyses revealed among the MDD group, higher loneliness was significantly associated with more positive parietal‐anterior cingulate cortex and cerebellum‐anterior cingulate cortex FC at rest.
					No significant main effect of loneliness across groups on rsFC.
**Sin et al., 2018** The relationships between brain structural changes and perceived loneliness in older adults suffering from late‐life depression	**52 older adults** (67.92 [5.0], > 60) *33 F/19 M* **3 depression groups**: single episode, multiple episodes, HC	Cross‐sectional	UCLA‐LS	GM volume (MRI) Whole‐brain analysis	Significant positive association between loneliness and GM volume within a large cluster (left putamen, left caudate, left globus pallidus) in single episode group, but negatively correlated for multiple episode group.
**Solé‐Padullés et al., 2022** No Association Between Loneliness, Episodic Memory and Hippocampal Volume Change in Young and Healthy Older Adults: A Longitudinal European Multicenter Study	**143 older adults BETULA sample** (63.97 [3.91], 60–85) *82 F/61 M* **1325 older adults BASE sample** (70.55[3.81], 60–86) *670F/655M*	Cross‐sectional & longitudinal	CES‐D & UCLA‐LS (harmonized loneliness scores across older adult cohorts)	GM volume, cortical thickness (MRI) ROI‐based analysis: hippocampus	No significant findings relating to loneliness and hippocampal volume or whole‐brain cortical thickness in either older adult samples.
**Spreng et al., 2020** The default network of the human brain is associated with perceived social isolation	**40,000 middle–older adults** (54.9, 40–69) *NA*	Cross‐sectional	Loneliness 1‐item—“Do you often feel lonely?”	GM + WM volume, FC (MRI, rsfMRI, DTI) Whole‐brain analysis	Loneliness was significantly positively associated with posterior superior temporal sulcus, left temporoparietal junction, left fusiform gyrus, right inferior temporal gyrus, right posterior parietal lobe, right dorsal anterior cingulate cortex volumes. Loneliness was significantly negatively associated with left dorsal anterior cingulate cortex, bilateral dorsolateral prefrontal cortex, right central operculum, right inferior parietal lobule, left retrosplenial cortex, bilateral inferior visual cortex volumes. Lonely individuals exhibited significantly greater within‐network coupling for DMN, and stronger FC between the DMN and other networks (limbic, dorsal attention, somatomotor). WM microstructural integrity was assessed for 48 WM tracts, with the strongest significant associations with loneliness involving 3 tracts that carry fibers of the fornix and originate from the hippocampus exhibiting greater microstructural integrity.
**Terracciano et al., 2025** Loneliness and Biomarkers of Alzheimer's Disease, Axonal Damage, and Astrogliosis: A Coordinated Analysis of Two Longitudinal Cohorts	**1263 middle–older adults** (59.69 [7.44], 46–80) *670 F/593 M*	Longitudinal	Loneliness 1‐item—“Do you often feel lonely?”	Aβ and tau (PET)	No significant associations between loneliness and Aβ or tau.
**van der Velpen et al., 2022** Social Health Is Associated With Structural Brain Changes in Older Adults: The Rotterdam Study	**3737 middle–older adults** (59.6 [8.0], 45–92) *2046 F/1691 M*	Longitudinal	CES‐D (1‐item).	WM volume, WMHs, TBV (MRI) ROI‐based analysis: hippocampus	Loneliness significantly associated with lower WM volume at baseline.
					No significant longitudinal associations between loneliness and WM volume. No significant associations between loneliness and hippocampal volume, TBV, GM volume, or WMHs.
**van der Velpen et al., 2023** Psychosocial health modifies associations between HPA‐axis function and brain structure in older age	**1259 middle‐older adults** (57.4 [6.40], > 40) *750 F/509 M*	Cross‐sectional	CES‐D (1‐item)	GM + WM volume, TBV, HPA axis negative feedback loop cortisol response (MRI, low‐dose DST) ROI‐based analysis: hippocampus, amygdala	No significant associations between loneliness and GM/WM volume, TBV, HPA axis cortisol response.
**van der Velpen et al., 2024b** Sex‐differences in the association of social health and marital status with blood‐based immune and neurodegeneration markers in a cohort of community‐dwelling older adults	**8375 middle–older adults** (65.7 [10.3], > 40) *4790 F/3585 M* **4099 neurodegeneration subset** (71.5 [7.3]) *2337 F/1762 M*	Cross‐sectional & longitudinal	CES‐D (1‐item)	Aβ, tau, NfL, systemic immune‐inflammation index (plasma).	No significant associations between loneliness and the immune system balance ratios or plasma neurodegeneration markers.
**Wong et al., 2016** Loneliness in late‐life depression: Structural and functional connectivity during affective processing	**54 older adults** **31 late‐life depression** (67.45 [5.43], > 60) *17 F/14 M* **23 HC** (67.13 [4.9], > 60) *14 F/9 M*	Cross‐sectional	UCLA‐LS	SC, FC, task‐evoked functional activity (DTI, fMRI task‐based). Task: affective processing, viewing affective stimuli	Loneliness was significantly associated with reduced SC within a subnetwork including various subcortical regions (middle temporal gyrus, amygdala, fusiform gyrus, inferior occipital gyrus, middle occipital gyrus, superior occipital gyrus, and calcarine fissure connecting to left globus pallidus, and edges from right calcarine fissure, lingual gyrus and amygdala connecting to superior frontal gyrus) which was evident in both groups. Loneliness in the LLD group was significantly negatively associated with task‐evoked functional activity within a large cluster involving the insula, putamen, globus pallidus and brain stem, along with frontal regions including inferior frontal gyrus, frontal pole, and frontal orbital cortex when processing negative stimuli, while the HC group exhibited a positive association. Loneliness in the LLD group significantly exhibited a positive association with FC across frontal regions, posterior cingulum, thalamus, some temporal and occipital areas when processing negative stimuli, with negative associations observed in HCs.
**Zajner et al., 2021** Loneliness is linked to specific subregional alterations in hippocampus‐default network covariation	**40,000 middle‐older adults** (54.9) *NA*	Cross‐sectional	Loneliness 1‐item—“Do you often feel lonely?”	GM volume (MRI) ROI‐based analysis: hippocampus and DMN subregions	Loneliness was significantly associated with reduced GM volume within dominant HC–DMN covariance modes, particularly involving hippocampal subregions (bilateral CA1 body, hippocampal fissure) and midline DMN areas (posterior cingulate cortex, retrosplenial cortex, dorsomedial prefrontal cortex), compared to non‐lonely participants.
**Zapater‐Fajari et al., 2025** Loneliness and biomarkers of brain pathology in people with subjective cognitive decline	**215 cognitively healthy older adults** (70.54 [0.26], 70) *114 F/101 M*	Cross‐sectional	CPRS 1‐item—“Do you feel lonely?”	WM signal abnormalities, Aβ, tau (MRI, CSF)	No significant associations between loneliness and Aβ, tau, or white matter signal abnormalities.
**Zhang et al., 2022** Perceived social isolation is correlated with brain structure and cognitive trajectory in Alzheimer's disease	**176 older adults** (78) *NA* **39 SCD** **53 MCI** **84 AD**	Cross‐sectional	Loneliness 1‐item—“Do you often feel lonely?”	GM volume (MRI) Whole‐brain analysis	Loneliness was significantly associated with lower GM volume in bilateral thalamus in SCD group, and lower volume in the left middle occipital gyrus, cerebellar vermal lobules I−V for the MCI group, compared to non‐lonely counterparts.
					No significant associations between loneliness and GM volume in AD group.
**Zhao et al., 2024** Association of Loneliness with Functional Connectivity MRI, Aβ PET, and Tau PET Neuroimaging Markers of Vulnerability for Alzheimer's Disease	**381 middle–older adults** **(fMRI sample)** (58 [10]) *188 F/193 M* **424 middle–older adults** **(PET sample)** (58 [10]) *213 F/211M*	Cross‐sectional	CES‐D 1‐item	Aβ, tau, FC (rsfMRI, PET)	No significant associations between loneliness and Aβ or tau deposition. No significant association between loneliness and functional connectivity.

Abbreviation: Aβ, amyloid beta; AD, Alzheimer's disease; ASL, arterial spin labeling; CA, cornu ammonis; CES‐D = Center for Epidemiologic Studies Depression Scale; CPRS, Comprehensive Psychopathological Rating Scale; CRP, C‐reactive protein; CSF = cerebrospinal fluid; DMN, default mode network; DST, dexamethasone suppression test; DTI, diffusion tensor imaging; FC, functional connectivity; fMRI, functional magnetic resonance imaging; GM, gray matter; HC, healthy control; HPA, hypothalamic–pituitary–adrenal; LLD, late‐life depression; MCI, mild cognitive impairment; MDD, major depressive disorder; MRI, magnetic resonance imaging; NfL, neurofilament light chain; PET, positron emission tomography; PFC, prefrontal cortex; RCT, randomized controlled trial; ROI, region of interest; rs, resting state; SC, structural connectivity; SCD, subjective cognitive decline; SES, socioeconomic status; TBV, total brain volume; TCV, total cranial volume; UCLA‐LS, University of California Los Angeles Loneliness Scale; VBM, voxel‐based morphometry; WM, white matter; WMHs, white matter hyperintensities.

**TABLE 3:** Studies that investigated both social isolation and loneliness.

Author/title	Sample Group/*n* (Mean age [SD]) *Female/male*	Study design	Loneliness and social isolation measures	Neurobiology measures	Key findings (Green = significant, red = non‐significant)
**D'Agostino et al., 2019** An fMRI study of loneliness in younger and older adults	**49 middle–older adults** (62.9 [6.1]) *30 F/19 M* **50 younger adults** (20.4 [2.0]) *26 F/24 M*	Cross‐sectional	UCLA‐LS Social Network Index—network size, number of high contact roles	Task‐evoked functional activity (fMRI task‐based) Whole‐brain & ROI‐based analyses: amygdala, ventral striatum Task: social affective task	No significant association between loneliness and functional activity within the amygdala or ventral striatum. No significant association between network size and functional activity within the amygdala or ventral striatum. No significant findings for whole‐brain analysis for either loneliness or network size.
**de Lange et al., 2021** Prominent health problems, socioeconomic deprivation, and higher brain age in lonely and isolated individuals: A population‐based study	**24,867 middle–older adults** (63.86 [7.51], 45–81) *12,445 F/12,422 M*	Cross‐sectional	Loneliness 1‐item—“Do you often feel lonely?” Social isolation: frequency of social contact, lives alone, frequency of social activities.	Brain age (XGBoost: compares cortical thickness, volume, surface area to standardized model, MRI)	Participants who were both socially isolated and lonely had a significantly higher brain age relative to chronological age compared to HCs. Higher social isolation scores alone were significantly associated with higher brain age.
					No significant main effect of loneliness on brain age.
**Duan et al., 2017** Empty‐nest‐related psychological distress is associated with progression of brain white matter lesions and cognitive impairment in the elderly	**66 empty nest single older adults** (69.41 [5.83], > 60) *37 F/29 M* **70 empty‐nest couples older adults** (70.46 [4.81], > 60) *35 F/35 M* **83 HC** (69.69 [5.75], > 60) *39 F/44 M*	Longitudinal	UCLA‐LS Empty nest (live alone, alone with partner)	WMH total, periventricular, deep volume (MRI)	Loneliness significantly positively associated with increased periventricular WMH, deep WMH, and total WMH volume across all groups. Both empty nest groups exhibited significantly greater periventricular WMH and total WMH volume compared to controls. Single empty nest group exhibited significantly greater deep WMH volume compared to controls. The percentage increase from baseline to 5‐year follow‐up in deep WMH volume for the single empty nest group was statistically higher than control and empty nest couples.
					No significant association between empty nest couples and deep WMH volume.
**Gao et al., 2024** The reciprocal associations between social deficits, social engagement, and inflammation: Longitudinal evidence comparing venous 2 samples and dried blood spots and mapping the modifying role of phenotypic and genotypic depression	**15,066 middle–older adults US** (66.1 [9.8], 52–100) *8737 F/6323 M* **10,290 middle–older adults UK** (66.9 [10.5], 50–99) *5623 F/4667 M*	Cross‐sectional & longitudinal	UCLA‐LS (3‐item) ELSA social isolation index: unmarried/not cohabiting (including living alone), non‐participation in any groups, clubs, or other social organizations, having less than monthly contact (face‐to‐face, telephone, written/e‐mail) with children, other family members, and friends	CRP (dried blood spots, venous samples)	In the US sample, loneliness at T1 (baseline) significantly predicted an increase in CRP at T2 (4 years). CRP had positive lagged effects on loneliness, CRP at T1 significantly predicted loneliness at T2 and T2 CRP predicted loneliness T3 (8 years). In the English sample, loneliness at T1 significantly predicted increased CRP at T2, and loneliness at T3 significantly predicted increased CRP at T4 (12–14 years). CRP at T1 significantly predicted an increase in loneliness at T2. Social isolation at T2 (4 years) significantly predicted CRP at T3 (8 years) in the English sample. CRP at T2 (4 years) significantly predicted social isolation at T3 (8 years). Cross‐sectional significant positive association between social isolation positively associated CRP in US sample only.
					No significant cross‐sectional loneliness findings for either cohort. No significant association between loneliness at T2/3 and CRP in US sample. No significant associations between social isolation predicting CRP in US sample. No significant association between social isolation and CRP at any other timepoint in English sample. No significant cross‐sectional social isolation association with CRP in English sample.
**Koyama et al., 2021** Interplay between social isolation and loneliness and chronic systemic inflammation during the COVID‐19 pandemic in Japan: Results from U‐CORONA study	**166 older adults** (65+)	Cross‐sectional	UCLA‐LS Number of daily social roles Categorical groups: non‐isolated–non‐lonely, isolated–non‐lonely, non‐isolated–lonely, isolated–lonely	CRP, neutrophil‐to‐lymphocyte ratio (blood samples)	No significant neuroinflammation findings in any older adult lonely/isolated groups.
**Noonan et al., 2023Δ** Home alone: A population neuroscience investigation of brain morphology substrates	**40,000 middle–older adults** (55.0 [7.5], 40–69) *20,800 F/19,200 M*	Cross‐sectional	Social isolation 1‐item—living alone or with others	GM volume (MRI). Whole‐brain analysis	Living alone was significantly associated with increased GM volume within IFG, mSTS, aSTS, middle temporal gyrus, ITG compared to those who live with others. Living alone was significantly associated with lower GM volume within pSTS, dorsomedial PFC compared to those who live with others. Living alone was significantly associated with increased volume within hippocampal subregions (positive volume effects—head: left CA1, right molecular layer, bilateral presubiculum, right CA2/3, right dentate gyrus; body: right presubiculum, left CA4). Living with others was significantly associated with increased volume within hippocampal subregions (negative volume effects—head: left dentate gyrus, left molecular layer; body: left presubiculum, right dentate gyrus). Various significant negative volume effects between living alone and amygdala nuclei (right central, right cortical, right corticoamygdaloid, left accessory basal nucleus). And a positive volume effect on right accessory basal nucleus.
**Shen et al., 2022Δ** Associations of Social Isolation and Loneliness With Later Dementia	**32,263 middle–older adults** (63.5 [7.5], 40–69) *16,777 F/15,486 M*	Cross‐sectional	Social isolation: frequency of social contact, lives alone, frequency of social activities— scoring 2–3 = socially isolated	GM volume (MRI) Whole‐brain analysis	Social isolation significantly associated with reduced GM volume within medial temporal lobe, hippocampus, middle temporal gyrus, fusiform gyrus, angular gyrus, ventromedial PFC, amygdala, thalamus.
**Shen et al., 2025Δ** Plasma proteomic signatures of social isolation and loneliness associated with morbidity and mortality	**42,062 middle–older adults** (56.4 [8.2], 40–69) *21,986 F/20,076 M*	Prospective	Social isolation: frequency of social contact, lives alone, frequency of social activities	Proteins (blood biomarkers)	175 proteins significantly associated with social isolation. Growth differentiation factor 15 strongest significant association with social isolation. Majority of significant proteins had a positive relationship with social isolation. These proteins were associated with insula, caudate, frontal cortex. Higher levels of ADM significantly related to lower GM volume in bilateral insula and left caudate.
**van der Velpen et al., 2024a∇** Determinants of social health trajectories during the COVID‐19 pandemic in older adults: the Rotterdam Study	**1720 middle–older adults** (68.7 [11.3]) *NA*	Longitudinal	CES‐D (1‐item), UCLA‐LS 3‐item	GM + WM volume, ICV, TBV (MRI) Segmentation‐derived global measures.	No significant associations between loneliness and neurobiology outcomes.

*Note*. **Δ** = also examined loneliness but used incorrect measure, **∇** = also examined social isolation but used incorrect measure. Abbreviations: ADM, adrenomedullin; aSTS, anterior superior temporal sulcus; CES‐D, Center for Epidemiologic Studies Depression Scale; CRP, C‐reactive protein; ELSA, English Longitudinal Study of Ageing; fMRI, functional magnetic resonance imaging; GM, gray matter; HC, healthy control; ICV, intracranial volume; IFG, inferior frontal gyrus; ITG, inferior temporal gyrus; MRI, magnetic resonance imaging; mSTS, middle superior temporal sulcus; PFC, prefrontal cortex; pSTS, posterior superior temporal sulcus; ROI, region of interest; TBV, total brain volume; UCLA‐LS, University of California Los Angeles Loneliness Scale; WM, white matter; WMH, white matter hyperintensity.

Given that relatively few studies directly examined social isolation and loneliness within the same analytical framework,^39^, ^40^, ^41^, ^42^, ^43^, ^44^, ^45^, ^46^, ^47^ these classifications were derived from patterns reported across studies rather than direct within‐study comparisons. For MRI findings, these classifications were applied irrespective of whether analyses were conducted using whole‐brain or region‐of‐interest approaches. Importantly, these categories should therefore be interpreted as reflecting trends in the existing literature rather than definitive evidence of neurobiological specificity, particularly given the variability in regions and spatial scales examined across studies. Accordingly, any apparent distinctions between the neurobiological correlates of social isolation and loneliness are tentative and should be interpreted with caution.

### 2.6 Quality assessment

Risk of bias assessment was conducted for the 42 included papers using the Appraisal tool for Cross‐Sectional Studies (AXIS)^48^ and Joanna Briggs Institute (JBI) critical appraisal tool for cohort studies (i.e., longitudinal, prospective, retrospective, matched cohort).^49^ Two reviewers (J.G. and N.J.) independently conducted risk assessments, and any disagreements were discussed and resolved.

The AXIS tool evaluates the quality of studies via 20 questions across different domains (introduction, methods, results, discussion, other) which are scored yes = 1, no = 0, toward the total score out of 20. If it was unclear whether the paper sufficiently addressed the criterion, it was flagged as “Don't know,” which was treated as a risk concern and scored as 0. While the AXIS tool does not provide a formal scoring system,^48^ we applied the quality rating levels used by other systematic reviews (e.g., Mehrabi et al.,^50^ Miri et al.,^51^ and Naeimabadi et al.^52^). For this review we reversed the categories to assess the risk of bias percentage: low risk (> 70%, ≥ 14 out of 20), moderate risk (60%–69.9%, 13–12 out of 20), high risk (0%–59.9%, ≤ 11 out of 20).

The JBI cohort study tool uses 11 questions to assess methodological quality across various domains, scored as yes = 1, no = 0, not applicable = subtract from total. We followed similar scoring protocols to previous studies (e.g., Goplen et al.^53^ and Kalniunas et al.,^54^). The risk of bias was considered high if < 49% of total responses were “yes,” moderate if total “yes” responses were between 50% and 69% of total score, and low if ≥ 70% of total responses were “yes.”

## 3 RESULTS

### 3.1 Details of included studies

This systematic review identified 149 unique articles from PubMed, PsycInfo, and Medline after deduplication. After title/abstract screening, 89 papers were deemed eligible for full‐text screening. Our search and screening yielded 42 papers that met our inclusion criteria. Of these, 7 examined social isolation,^55^, ^56^, ^57^, ^58^, ^59^, ^60^, ^61^ 26 loneliness,^62^, ^63^, ^64^, ^65^, ^66^, ^67^, ^68^, ^69^, ^70^, ^71^, ^72^, ^73^, ^74^, ^75^, ^76^, ^77^, ^78^, ^79^, ^80^, ^81^, ^82^, ^83^, ^84^, ^85^, ^86^, ^87^ and 9 investigated both^39^, ^40^, ^41^, ^42^, ^43^, ^44^, ^45^, ^46^, ^47^ (see Tables 1, 2, 3). Of the nine that investigated both, three used an inaccurate measure of loneliness^44^, ^45^, ^46^ and one an inaccurate social isolation measure.^47^ In line with the eligibility criteria, only findings relating to the correctly operationalized construct were included in the results.

### 3.2 Methodological risk of bias

Upon assessment, 41 studies were deemed to overall have low risk of bias, and 1 study was considered to have moderate risk;^79^ see Supplementary Material. However, the majority of studies revealed moderate risk of bias within the results domain, particularly regarding not accounting for non‐response bias or response rates. Additionally, a few studies exhibited moderate risk of bias for their methodologies, specifically not justifying same sizes, not using valid/reliable measures for exposure variables, not addressing/categorizing non‐responders, and incomplete follow‐up (for cohort studies).

### 3.3 Age

Of the included papers, 20 combined middle‐aged and older adult participants into a single sample for analysis, 19 included older adults only (aged 60–65+ years), and 3 were specifically middle‐aged adults (< 65 years).

### 3.4 Sample characteristics

Most papers investigated cognitively healthy middle‐older adults (*n* = 35), two studies included an AD subsample,^74^, ^86^ one a mild cognitive impairment group,^86^ and four depression/major depressive disorder.^68^, ^75^, ^76^, ^83^

### 3.5 Study designs

Twenty‐seven studies used a cross‐sectional design, seven were longitudinal, one retrospective, one prospective, one randomized controlled trial (RCT), one matched cohort, one matched case–control, and three used both cross‐sectional and longitudinal analyses.

### 3.6 Measures

#### 3.6.1 Social isolation and loneliness measures

Table 4 summarizes the various social isolation and loneliness measures used across the included studies, demonstrating considerable heterogeneity in operationalizations.

**TABLE 4:** Social isolation and loneliness measures used across studies.

Social construct	Measure/instrument	N	Validation status
Social isolation	Lubben Social Network Scale	3	Validated to measure perceived quality/availability of social ties within social network
	Social Network Index	2	Validated to measure social integration
	UK Biobank items (frequency of social contact, living alone, frequency of social activities)	3	Not validated
	Living alone	2	Not validated
	Frequency of contact	1	Not validated
	Number of social roles	1	Not validated
	Living alone & frequency of contact	2	Not validated
	Living alone & no close relationships	1	Not validated
	English Longitudinal Study of Ageing social isolation index	1	Empirically validated in cohort studies
			
Loneliness	University of California Los Angeles Loneliness Scale	19	Validated
	De Jong Loneliness Scale	2	Validated
	Center for Epidemiologic Studies Depression Scale (single‐item: “*I felt lonely*)”	8	Derived from validated depression scale; not validated for loneliness
	loneliness single‐item (“I felt lonely”)		
	“Do you often feel lonely”	5	Not validated
	Comprehensive Psychopathological Rating Scale (single‐item: “*Do you feel lonely?*”)	1	Derived from validated measure of psychiatric symptoms severity; not validated for loneliness
	loneliness single‐item (“Do you feel lonely?”)		
	UCLA‐LS & CES‐D	3	See above

Abbreviations: CES‐D, Center for Epidemiologic Studies Depression Scale; UCLA‐LS, University of California Los Angeles Loneliness Scale.

#### 3.6.2 Neurobiological outcome measures

Neuroimaging techniques from most to least used were MRI (*n* = 23), fMRI (*n* = 9; task based = 3, resting state = 4, both = 1), PET (*n* = 6), and DTI (*n* = 3). See Table 5 for a detailed breakdown of neurobiological outcome measures used across social isolation and loneliness studies.

**TABLE 5:** Number of social isolation and loneliness studies using various neurobiological outcome measures.

Neurobiological outcome measure	Social isolation	Loneliness
Gray matter volume	(*n* = 5)	(*n* = 11)
Whole‐brain	(*n* = 3)	(*n* = 3)
ROI based	(*n* = 2)	(*n* = 7)
White matter hyperintensity/lesion volume (signal abnormalities)	(*n* = 3)	(*n* = 6)
Total brain volume	(*n* = 1)	(*n* = 4)
Cortical thickness	(*n* = 1)	(*n* = 2)
Functional activity	(*n* = 1)	(*n* = 3)
C‐reactive protein concentration	(*n* = 3)	(*n* = 2)
Neutrophil‐to‐lymphocyte ratio	(*n* = 1)	(*n* = 1)
Amyloid beta burden	(*n* = 2)	(*n* = 6)
Tau burden	(*n* = 1)	(*n* = 5)
Morphometry‐derived brain age (cortical thickness, volume, surface area)	(*n* = 1)	(*n* = 1)
White matter volume	–	(*n* = 5)
White matter tract integrity	–	(*n* = 1)
Functional connectivity	–	(*n* = 7)
Structural connectivity	–	(*n* = 1)
Total cranial volume	–	(*n* = 1)
Intracranial volume	–	(*n* = 1)
Fibrinogen concentration	(*n* = 1)	–
Albumin concentration	(*n* = 1)	–
Inflammatory proteomic markers	(*n* = 1)	–

Abbreviation: ROI, region of interest.

### 3.7 Covariates

The included studies adjusted for a range of demographic, social and socioeconomic, health‐related, and clinical covariates. Across the 15 studies examining social isolation, demographic covariates most commonly included were age (*n* = 12) and sex (*n* = 12), while social and socioeconomic covariates included education (*n* = 13), income (*n* = 7), employment/occupational status (*n* = 4), and marital status (*n* = 2). Health‐related covariates included body mass index (BMI; *n* = 10), smoking (*n* = 10), alcohol (*n* = 10), exercise (*n* = 7), cognition (*n* = 3). Clinical conditions commonly controlled for were diabetes (*n* = 8), depression/mental health symptoms (*n* = 7), hypertension (*n* = 7), apolipoprotein E (*APOE*) ε4 status (*n* = 3). Note, two of the studies did not report the covariates included in their analyses.

Across the 32 loneliness studies, demographic covariates most commonly included were age (*n* = 22) and sex (*n* = 21). The social and socioeconomic covariates included were education (*n* = 17), social network size (*n* = 3), income (*n* = 3), socioeconomic status (*n* = 2), and employment status (*n* = 2). Health‐related covariates included smoking (*n* = 9), alcohol (*n* = 9), BMI (*n* = 9), exercise (*n* = 4), and cognition/memory (*n* = 4). Clinical conditions commonly controlled for were depression symptoms (*n* = 11), *APOE* ε4 status (*n* = 6), anxiety (*n* = 5), diabetes (*n* = 5), and hypertension (*n* = 5). Seven of the studies did not report the covariates adjusted for in their analyses.

### 3.8 Statistical reporting of associations between social health and neurobiology

Across the included studies, quantitative measures of significant associations among social isolation, loneliness, and neurobiological outcomes were inconsistently reported. Of the 42 included studies, 11 reported extractable quantitative effect estimates for significant associations between social isolation or loneliness and neurobiology outcomes, while the remaining 31 did not provide extractable effect estimates for their significant findings. When reported, these statistical measures used varied considerably, including correlation coefficients,^39^, ^72^, ^76^, ^78^, ^83^ standardized regression coefficients,^42^, ^65^, ^66^ and effect sizes.^40^, ^64^, ^70^ This heterogeneity in statistical reporting limits comparability across studies and precludes formal meta‐analysis.

### 3.9 Exclusive neurobiological patterns associated with social isolation and loneliness

#### 3.9.1 Social isolation: gray matter structures

In temporal structures, there was a small degree of consensus, with three of six social isolation studies exploring gray matter (GM) volume (two whole‐brain, one region of interest [ROI] based) reporting significant associations between social isolation and the middle temporal gyrus (MTG). A cross‐sectional study measuring social isolation in older adults found that social contact several times a month with non‐cohabitating friends and relatives was associated with lower MTG volume.^57^ Another cross‐sectional study of middle–older adults (*N *= 32,263) found social isolation was significantly associated with reduced GM volume within the MTG.^45^ In contrast, Noonan et al.’s^44^ cross‐sectional study revealed living alone was associated with greater GM volume within the MTG in middle–older adults (*N* = ≈ 40,000). Additionally, two of these studies also uncovered a significant association between social isolation and lower GM volume within the temporal lobe^57^ and medial temporal lobe.^45^

Looking at other GM structures, low social contact (seldom/several times a month) was also associated with reduced volume within the occipital lobe and cingulum, while seldom having social contact specifically was associated with lower volume within the transverse temporal gyrus and rostal anterior cingulate gyrus.^57^ Another study identified a significant association between social isolation and reduced GM volume within the angular gyrus and ventromedial prefrontal cortex.^45^ Moreover, middle–older adults who lived alone exhibited greater GM volume within the inferior frontal gyrus, middle superior temporal sulcus, and anterior superior temporal sulcus, compared to those living with others.^44^

Using morphometry‐derived brain age, de Lange et al.^40^ estimated the brain age of lonely (single‐item question) and isolated (frequency of social contact, lives alone, frequency of social activities) middle–older adults by comparing various structural MRI features (i.e., cortical thickness, volume, surface area) to a standardized normative model, and found participants who were both socially isolated and lonely had a higher brain age relative to chronological age compared to healthy controls (HCs). Additionally, higher social isolation scores alone were associated with higher brain age compared to chronological; however, loneliness alone was not significantly associated.

#### 3.9.2 Loneliness: gray matter structures

Two cross‐sectional studies reported significant associations between loneliness and globus pallidus volume in middle‐aged to older adults. Lee et al.^71^ found two loneliness measures were associated with reduced volume within the globus pallidus, specifically the left for UCLA‐LS and bilaterally for Center for Epidemiological Studies Depression Scale (CES‐D). Sin et al.^76^ reported loneliness (UCLA‐LS) was positively associated with left globus pallidus volume in older adults (*N *= 52) experiencing a single episode of depression, but negatively correlated for those who experienced multiple depressive episodes.

Two cross‐sectional studies reported significant associations between loneliness and putamen volume; however, the direction of these relationships was contrasting. Sin et al.^76^ reported loneliness was positively associated with putamen volume in the single depression episode group but negatively correlated for the multiple episode group, while Lee et al.^71^ found loneliness measured by the CES‐D, but not the UCLA‐LS, displayed significant GM volume reductions within the putamen.

The exclusive neurobiological patterns associated with social isolation and loneliness are presented in Figure 2.

**FIGURE 2:**
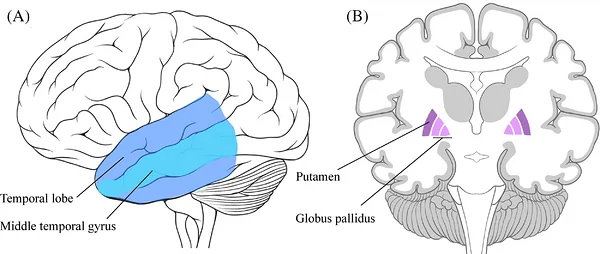
Gray matter cortical and subcortical regions showing exclusive patterns of association with social isolation or loneliness. Highlighted regions indicate exclusive patterns of association with each construct across the literature, replicated in ≥ 2 studies. A, Social isolation. B, Loneliness.

Looking at other GM structures, a UK Biobank study of middle–older adults (*N* = ≈ 40,000) found loneliness (single‐item question) was positively associated with volume in the left temporoparietal junction, right posterior parietal lobe, and right dorsal anterior cingulate cortex.^78^ Another study found loneliness (single‐item question) in mild cognitive impairment (MCI), but not in Alzheimer's disease (AD) was significantly associated with lower volume within the left middle occipital gyrus and cerebellar vermal lobules I to V compared to non‐lonely counterparts. A comparison of middle–older (*n *= 45) and younger adults (*n *= 44) found middle–older adults reporting high loneliness (UCLA‐LS) exhibited greater cerebral blood flow (CBF) within the left supramarginal gyrus compared to younger adults reporting high loneliness.^63^ Lee et al.^71^ found CES‐D loneliness, but not UCLA‐LS, displayed significant reductions in cerebral and subcortical GM volume. Additionally, loneliness (single‐item question) assessed in ≈ 40,000 middle–older adults was associated with reduced volume within midline default mode network (DMN) regions, particularly the posterior cingulate cortex and retrosplenial cortex, compared to non‐lonely participants,^84^ while loneliness in participants only experiencing a single depressive episode was positively associated with left caudate volume and negatively associated with loneliness for participants experiencing multiple depressive episodes.^76^

#### 3.9.3 Loneliness: functional activity findings

Wong et al.^83^ investigated the relationship between loneliness (UCLA‐LS) and task‐evoked functional activity (FA), and found loneliness in the late‐life depression group (*n *= 31) was negatively associated with task‐evoked FA within a large cluster involving the insula, putamen, globus pallidus, and brain stem, along with frontal regions including inferior frontal gyrus, frontal pole, and frontal orbital cortex when processing negative stimuli, while the HC group (*n *= 23) exhibited a positive association between loneliness and these regions. D'Agostino et al.^39^ also investigated the differing associations between loneliness (UCLA‐LS) and social isolation (Social Network Index [SNI]) on FA within the amygdala and ventral striatum, alongside whole‐brain analysis; however, they did not uncover any significant associations.

### 3.10 Shared neurobiological patterns across social isolation and loneliness

#### 3.10.1 Gray matter structures shared between social isolation and loneliness

The strongest overlap across the included studies was observed for the hippocampus, with five social isolation studies^44^, ^45^, ^57^, ^59^, ^60^ and three loneliness studies^66^, ^71^, ^84^ reporting significant associations (Figure 3).

**FIGURE 3:**
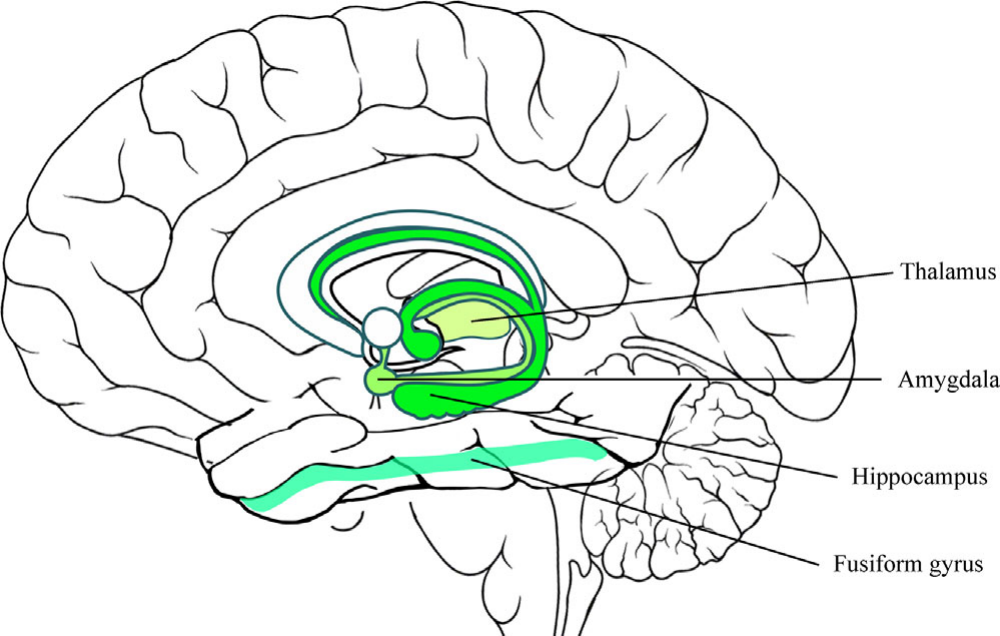
Gray matter cortical and subcortical regions showing shared patterns of association with social isolation and loneliness. Highlighted regions indicate structures reported in ≥ 3 significant associations across the included studies, with evidence derived from both social isolation and loneliness. Note: Brain templates used in Figures 2−3 were adapted from Wikimedia Commons images by Patrick J. Lynch, medical illustrator (CC BY 2.5), Hariadhi (CC BY‐SA 4.0), and Ryan Kissinger/NIAID (NIH BioArt; public domain).

In particular, four of the social isolation studies observed a reduction in hippocampal volume in socially isolated participants^45^, ^57^, ^59^, ^60^ and one reported bidirectional volumetric associations within various hippocampal head (+ left CA1, right molecular layer, bilateral presubiculum, right CA2/3, right dentate gyrus; − left dentate gyrus, left molecular layer) and body (+ right presubiculum, left CA4; − left presubiculum, right dentate gyrus) subregions compared to cohabitating middle–older adults.^44^ Notably, Murayama et al.^60^ compared different operationalizations of social isolation (i.e., frequency of social contact and living alone), reporting only low‐frequency social contact was significantly associated with reduced hippocampal volume over time.

Similar outcomes were observed for loneliness, with higher UCLA‐LS scores associated with reduced volume within the anterior hippocampus and left posterior parahippocampal gyrus,^66^ while Lee et al.^71^ found CES‐D loneliness was associated with reduced volume within the right hippocampus, but not UCLA‐LS loneliness. Moreover, Zajner et al.^84^ found loneliness (single‐item question) was associated with reduced volume within hippocampal subregions (bilateral CA1 body, hippocampal fissure) compared to non‐lonely participants.

In contrast, six studies with a total *N *= 8717 failed to uncover any significant hippocampal associations with loneliness measured via the single‐item CES‐D,^73^, ^80^, ^81^ 3‐item UCLA‐LS,^47^ full UCLA‐LS,^67^ and harmonized loneliness scores using both the CES‐D and UCLA‐LS.^77^

The amygdala also demonstrated mild convergence across the included papers, with three studies reporting significant associations with social isolation^44^, ^45^, ^57^ and one with loneliness,^66^ all of which were cross‐sectional. Of the three studies, two reported an association between social isolation and reduced amygdala volume,^45^, ^57^ while one study identified bidirectional associations between living alone and amygdala nuclei volume (− right central, right cortical, right corticoamygdaloid, left accessory basal nucleus; + right accessory basal nucleus) compared to cohabitating middle–older adults.^44^

Similarly, higher UCLA‐LS scores were associated with reduced amygdala volume in cognitively healthy older adults.^66^ This is contrasted by a RCT that reported changes in loneliness after 6‐month RCT had no significant impact on amygdala volume.^67^

Two studies reported significant associations between loneliness and reduced volume within the thalamus,^71^, ^86^ while one study uncovered a similar association with social isolation.^45^ Notably, only the subjective cognitive decline subgroup was associated with reduced bilateral thalamus volume compared to their non‐lonely counterparts, not observed for mild cognitive impairment or AD subgroups.^86^

Two studies reported significant associations between social isolation and reduced volume within the fusiform gyrus,^45^ specifically social contact several times a month.^57^ In contrast, loneliness was associated with greater volume in left fusiform gyrus.^78^

One study using the UCLA‐LS found loneliness scores were negatively correlated with entorhinal cortex volume in cognitively healthy older adults.^66^ A similar pattern was observed for social isolation, with infrequent social contact associated with lower volume within the entorhinal cortex.^57^

Middle–older adults reporting high loneliness exhibited greater CBF within the bilateral superior temporal gyrus compared to younger adults reporting high loneliness,^63^ while low social contact (seldom/several times a month) was associated with smaller volume within the superior temporal gyrus.^57^

Loneliness was positively associated with volume in the right inferior temporal gyrus,^78^ while infrequent social contact was associated with reduced volume.^57^

Loneliness was associated with greater volume within the posterior superior temporal sulcus,^78^ while people who live alone exhibited lower volume compared to those living with others.^44^

Middle–older adults who lived alone exhibited lower volume within the dorsomedial prefrontal cortex compared to those cohabitating.^44^ Similarly, loneliness was associated with reduced dorsomedial prefrontal cortex volume compared to non‐lonely participants.^84^

The findings detailed above are contrasted by studies reporting null results. For social isolation, one study did not report any significant longitudinal associations between social isolation (living alone/frequency of social contact) and changes in total GM volume.^60^ Similarly, several studies failed to uncover significant associations between loneliness and GM volume within any of their ROIs,^47^, ^80^, ^81^ all of which used the CES‐D, while van der Velpen et al.^47^ included both the CES‐D and 3‐item UCLA‐LS measures. Additionally, a main effect of loneliness on CBF in middle–older adults was not uncovered.^63^

#### 3.10.2 White matter characteristics shared between social isolation and loneliness

Social isolation and loneliness have both somewhat consistently been associated with increased of white matter (WM) hyperintensity/lesion volume. One social isolation study found living alone and empty nesting with a spouse both exhibited greater total and periventricular WM hyperintensities (WMHs) compared to HCs.^41^ Distinct characteristics between these groups were also revealed, as living alone was associated with deep WMHs, and a statistically higher increase in deep WMH volume from baseline to 5‐year follow‐up compared to empty nesting couples and HCs. Smaller social networks in older adulthood were likewise related to greater WMHs, with smaller family networks but not friendship networks associated with severe WMHs.^55^ Similarly, low‐frequency social contact was linked to increased WM lesion volume.^57^

Loneliness has also been associated with increased WMHs,^72^, ^73^ specifically periventricular, deep, and total WMH volume.^41^ Loneliness measured by both the UCLA‐LS and CES‐D were both associated with larger WMH volumes compared to non‐lonely counterparts.^71^ However, these findings are contrasted by van der Velpen et al.^80^ who did not uncover any significant findings relating to loneliness and WMH volume. Additionally, Zapater‐Fajari et al.^85^ investigated the association between loneliness and WM signal abnormalities; however, they did not uncover any significant results.

#### 3.10.3 Total brain volume

Both infrequent social contact^57^ and loneliness measured by the CES‐D, but not the UCLA‐LS,^71^ were associated with lower total brain volume (TBV). In contrast, three studies reported no association between loneliness and TBV.^47^, ^80^, ^81^

#### 3.10.4 Neuroinflammatory markers shared between social isolation and loneliness

Social isolation was associated with higher levels of inflammation measured by C‐reactive protein (CRP) in older men but not older women.^61^ A bidirectional longitudinal study of social isolation (SNI), loneliness (3‐item UCLA‐LS), and CRP concentration found social isolation at 4‐year follow‐up was associated with CRP concentration at 8‐year follow‐up in the UK sample, and the inverse relationship was observed within the US sample.^42^ Baseline loneliness predicted CRP concentration at 4‐year follow‐up in both samples, and the UK cohort found loneliness at 8‐year follow‐up predicted CRP concentration at 12‐ to 14‐year follow‐up. Baseline CRP predicted loneliness at 4‐year follow‐up in both cohorts, while CRP at 4‐year follow‐up predicted loneliness at 8‐year follow‐up in the US sample only.

Koyama et al.^43^ examined the interplay between social isolation (number of daily social roles) and loneliness (UCLA‐LS) on chronic systemic inflammation (CRP, neutrophil‐to‐lymphocyte ratio); however, they did not uncover any significant results.

#### 3.10.5 Neurodegenerative markers shared between social isolation and loneliness

A cross‐sectional study investigating the association between level of social isolation risk (Lubben Social Network Scale [LSNS]) and Aβ burden (PET) within middle‐aged adults found those at low social isolation risk were more likely to exhibit elevated Aβ compared to participants at moderate risk, while the merged isolated/high‐risk group showed no association.^56^ Another study examining social isolation (living alone or having no close relatives/friends/neighbors) and Aβ burden (CSF) found no significant association.^58^ A significant, albeit weak, association between loneliness (UCLA‐LS) and cortical Aβ (PET) was identified in cognitively normal older adults by Kehrer‐Dunlap et al.^69^ Another PET study found loneliness (3‐item UCLA‐LS) was related to higher cortical Aβ burden in cognitively normal older adults; moreover, amyloid‐positive participants (*N* = 79) were more likely to experience loneliness than those who were amyloid negative.^65^

Hou et al.^58^ also examined the association between social isolation and tau burden, finding non‐isolated participants exhibited significantly lower tau and p‐tau compared to those who were socially isolated. d'Oleire Uquillas et al.^64^ explored the association between tau pathology within the entorhinal and inferior temporal cortices and loneliness (3‐item UCLA‐LS, analyzed as a dependent variable) and found tau pathology within the right entorhinal cortex was associated with greater loneliness, remaining significant after adjusting for social network, depression, anxiety, memory, and socioeconomic status. Exploratory analysis revealed an additional positive correlation between tau within the right fusiform gyrus and greater loneliness; however, no significant findings were uncovered within the left entorhinal cortex or inferior temporal cortex. This is contrasted by studies using PET,^79^, ^87^ CSF,^85^ and plasma‐based analyses^82^ that did not uncover any significant findings relating to loneliness and Aβ or tau burden.

### 3.11 Non‐comparable neurobiology findings

There was a large degree of heterogeneity in the neurobiological outcomes explored across the included papers, with some loneliness and social isolation studies examining discrete features of neurobiology (see Table 5). Consequently, these results are not directly comparable and highlight a broader issue within the social isolation and loneliness discipline. As these outcomes did not allow for a meaningful synthesis across social constructs, they are not reported in detail in the this section. Instead, these non‐comparable neurobiological findings are presented in Tables 1, 2, and 3 for completeness and transparency.

### 3.12 Summary of results

In summary, the results identified neurobiological patterns reported exclusively for social isolation and loneliness, as well as patterns shared between the two. However, a considerable proportion of findings could not be directly compared, as studies frequently examined different neurobiological outcomes. Convergence across exclusive and shared findings was most evident for GM volumetric measures, likely as this neurobiological outcome was used the most across the included studies (see Table 5). Specifically, the most commonly reported exclusive pattern across the literature was observed between social isolation and GM volume within temporal lobe structures (i.e., middle temporal gyrus), with three independent studies reporting significant findings. Moreover, loneliness demonstrated an exclusive pattern of association with GM volume within the putamen and globus pallidus. The greatest shared pattern across social isolation and loneliness was observed for GM volume within the hippocampus, with eight studies reporting significant associations, followed by the amygdala (*n* = 4) and thalamus (*n* = 3). Additionally, larger WMH/lesion volume was observed for both social isolation and loneliness, reported by five studies. Social isolation and loneliness also exhibited similar patterns of heightened CRP concentration.

## 4 DISCUSSION

To our knowledge, this is the first systematic review exploring the neurobiological correlates underpinning both social isolation and loneliness in middle–older adults. From synthesizing the results across the 42 studies, both exclusive and shared neurobiological patterns associated with social isolation and loneliness were identified. The greatest shared pattern between social isolation and loneliness involved GM volume within various limbic structures (i.e., hippocampus, amygdala, thalamus), whereas the most commonly reported exclusive pattern was observed for social isolation and GM volume within temporal lobe structures. As the studies differed in the GM regions examined and analytic approaches used, these patterns should be interpreted as trends within the existing literature rather than definitive neurobiological distinctions. Furthermore, these findings are limited by substantial methodological heterogeneity evident across three key domains, operationalization of social isolation and loneliness, neurobiological outcomes, and statistical reporting, which will be discussed.

### 4.1 Interpreting neurobiological patterns shared by social isolation and loneliness

Across the literature, the patterns of associations suggest a large degree of overlap in GM structures implicated in both social isolation and loneliness, including the hippocampus, amygdala, thalamus, entorhinal cortex, fusiform gyrus, superior temporal gyrus, inferior temporal gyrus, posterior superior temporal sulcus, and dorsomedial prefrontal cortex. Many of these regions are central to the social brain theoretical framework,^18^ which posits that specific GM regions display heightened activity when performing social tasks (i.e., mentalizing, empathy, face perception) during MRI scanning. The three GM structures showing the largest degree of overlap between social isolation and loneliness, based on the number of studies reporting significant associations, were the hippocampus (*n* = 8), amygdala (*n* = 4), and thalamus (*n* = 3). These structures form part of the limbic system, which is responsible for various social cognition functions, including social cue recognition,^88^ detecting social/emotional salient stimuli,^89^ social memory,^90^ and mapping social information.^91^ Therefore, it is not unexpected that these particular limbic structures have been implicated in social isolation and loneliness, as social cognitive functioning is inhibited or less frequently used. However, as the majority of these studies used cross‐sectional designs (7/9), these findings should be interpreted with caution as temporal relationships cannot be established.

Lonely and socially isolated middle–older adults also exhibited increased WMH/lesion volume,^41^, ^57^, ^71^, ^72^, ^73^ suggesting poor social health may be associated with compromised WM structural integrity. The social stress accompanied by persistent loneliness may engage biological stress responses such as hypothalamic–pituitary–adrenal axis activation, which has been linked to WMH burden.^92^ Additionally, social networks have been proposed to exert protective effects on brain health.^55^ Consequently, social environments characterized by low social engagement or inadequate stimulation may contribute to detrimental changes in WM structure.^41^ Again, this evidence has been predominantly derived from cross‐sectional designs (4/6), limiting conclusions regarding temporality.

Many of these overlapping neurobiological patterns associated with social isolation and loneliness have also been implicated in dementia research, displaying vulnerability to neurodegenerative patterns of atrophy. In particular, the hippocampus and amygdala are strongly associated with dementia (e.g., Couvey‐Duchesne et al.^93^ and Nakazawa et al.^94^), with reduced hippocampal volume being a significant predictor of long‐term dementia risk and neurodegenerative progression (e.g., Manco et al.^95^ and Gentreau et al.^96^), and amygdala atrophy a key feature of AD.^97^ Moreover, WMHs have been associated with an increased risk of dementia;^98^ notably, a meta‐analysis revealed baseline WMHs contributed to a 73% elevated risk of vascular dementia and 25% risk of AD.^99^

### 4.2 Interpreting exclusive neurobiological patterns of social isolation

The most commonly reported exclusive neurobiological pattern was the association between social isolation and volume within temporal structures, which was not reported for loneliness. Two studies reported significantly smaller GM volume within the MTG and broader temporal lobe regions in socially isolated middle–older adults,^45^, ^57^ whereas one study observed larger MTG volume.^44^ Notably, all three studies used single‐item indicators to operationalize social isolation (e.g., frequency of social contact, frequency of social activities, and living alone), rather than established multi‐item validated scales (e.g., LSNS, SNI), which more commonly reported non‐significant results.^39^, ^56^, ^59^, ^61^ These single‐item indicators may capture more objective aspects of social isolation, whereas multi‐item scales often incorporate subjective elements (e.g., perceived closeness, perceived quality and availability of social ties), although the small number of studies limits firm conclusions and further research is needed to clarify these discrepancies. Moreover, these findings highlight variability in the operationalization of social isolation, particularly between single‐item indicators and validated scales, with broader methodological considerations discussed further below.

The temporal lobe comprises multiple subregions that collectively support social cognition and affective processing; these functions include person memory, perceiving social stimuli, theory of mind, and socio‐emotional integration (e.g., Wang and Olson;^18^ Olson et al.;^100^ and Bryant and Schurz^101^). The MTG lies within the temporal lobe on the lateral surface. Converging evidence indicates that the MTG serves a key role in various social cognitive functions, including social semantic and theory of mind‐related processing (i.e., inferring mental states and behaviors of others),^102^ social processing,^103^ and empathy‐based interpersonal emotion regulation.^104^ Hence, in the context of social isolation and the reported findings of Hirabayashi et al.,^57^ and Shen et al.,^45^ lower MTG volume may arise from reduced use due to limited social interaction (e.g., Lammer et al.^59^). While Noonan et al.'s^44^ observed increase in volume may reflect a compensatory mechanism, whereby living alone increases reliance on internally driven social‐cognitive processes (e.g., mentalizing and semantic processes) supported by the MTG, resulting in internal neuroplastic adaptations to offset reduced social interaction when socially isolated.^9^ Additionally, studies have reported that the MTG is particularly susceptible to aging,^102^, ^105^ exhibiting declined cortical activity and delayed activation in older adults, making it particularly vulnerable to social isolation–related dementia risk.

The MTG has also exhibited high vulnerability toward early‐stage AD,^106^ it being considered one of the first temporal sites affected in AD via atrophy, abnormal gene expression, plaques, and tangles, which can predict progression from typical aging to AD (e.g., Convit et al.^107^ and Piras et al.^108^). Furthermore, as the significant associations with MTG volume were reported exclusively for social isolation, this region may provide preliminary insight into the neurobiological mechanisms linking social isolation to dementia risk. However, as these three studies were all cross‐sectional designs, stronger evidence from longitudinal studies is required to establish temporal relationships.

### 4.3 Interpreting exclusive neurobiological patterns of loneliness

Loneliness was associated with GM volume within the putamen and globus pallidus, which was not reported for social isolation.^71^, ^76^ The putamen and globus pallidus form the lentiform nucleus located within the basal ganglia; however, while anatomically related, these regions are structurally and functionally distinct.^109^ The putamen's primary functions are regulating voluntary motor control, stimulus–action–reward associations, and procedural learning,^110^ and has exhibited involvement in various social cognitive processes, such as intuitive social action^111^ and empathy.^112^ The globus pallidus similarly contributes to controlling voluntary movement, acting as a key relay and output structure within the basal ganglia circuits,^113^ and is also involved in motivation, reward valuation, and action–inhibition, which support socially guided behaviors (e.g., Rossi et al.^114^ and Gu et al.^115^).

The basal ganglia's subcortical structures have been increasingly implicated in neurodegeneration and dementia, with studies reporting structural, functional, and vascular aberrances.^116^ Putamen volume is considered age dependent,^117^ with numerous studies reporting decreased GM volume in AD (e.g., de Jong et al.^118^ and Möller et al.^119^) and frontotemporal dementia (FTD) cohorts.^119^, ^120^ Moreover, smaller globus pallidus volume has been observed in people with FTD compared to AD.^119^ Other biomarkers of neurodegeneration have been investigated in reference to the globus pallidus and putamen. One study reported greater blood‐brain barrier permeability within the globus pallidus in AD participants, reflected by higher Ktrans values compared to HCs and those with dementia with Lewy bodies (DLB).^121^ Furthermore, a meta‐analysis examining iron accumulation across cortical (i.e., thalamus, hippocampus) and subcortical regions (i.e., basal ganglia) revealed significantly elevated iron levels in people with AD compared to HCs, with the most pronounced effects observed in the putamen.^122^ These findings provide preliminary evidence that the putamen and globus pallidus may be relevant to neurodegenerative processes implicated in dementia; however, as these were cross‐sectional studies this should be interpreted with caution.

Overall, evidence pertaining to the neurobiological patterns of social isolation and loneliness remains mixed and inconclusive, with several studies reporting predominantly non‐significant associations across structural, functional, neurodegenerative, and neuroinflammatory outcomes (e.g., D'Agostino et al.^39^ and Hou et al.^58^). Interestingly, a number of these studies used single‐item questions to operationalize loneliness,^79^, ^80^, ^81^, ^82^, ^85^, ^87^ which aligns with ongoing concerns surrounding the sensitivity of single‐item questions in accurately capturing the experience of loneliness. ^5^, ^123^, ^124^ Future studies are encouraged to use validated multi‐question measures such as the UCLA‐LS,^125^ which has undergone rigorous validity and reliability evaluations (e.g., Alsubheen et al.^126^). Additionally, as the bulk of this evidence was obtained from cross‐sectional studies, it is unclear whether these neurobiological patterns persist or change over time. Stronger longitudinal study designs should be used to clarify these relationships.

### 4.4 Methodological considerations and future directions

There was a high degree of heterogeneity across the included studies in relation to the neurobiological outcomes examined and neuroimaging methodologies used. Consequently, the bulk of the findings pertaining to social isolation and loneliness cannot be directly compared. Studies varied considerably in MRI modalities examined (i.e., structural, functional, WM characteristics), as well as the spatial scales (i.e., whole brain, ROI based), and specific regions/subnetworks selected for investigation. This is particularly evident for fMRI and WM characteristics, as only one social isolation study used fMRI juxtaposed to eight loneliness fMRI studies. Additionally, no social isolation studies investigated broader WM volume or tract integrity, despite six loneliness studies doing so. As a result, the extent to which functional and WM outcomes represent shared or exclusive neurobiological correlates of social isolation and loneliness remains unclear. As such, the present review cannot infer definitive neurobiological specificity for either social isolation or loneliness, and the identified patterns should therefore be interpreted as trends within the literature rather than conclusive distinction. This highlights the need for future studies to investigate these outcomes using more consistent neuroimaging approaches.

Heterogeneity was also evident across the various operationalizations of social isolation and loneliness, ranging from single‐item measures (e.g., CES‐D, “*Do you feel lonely?*”; frequency of social contact) to validated tools (e.g., UCLA‐LS, LSNS). Studies also differed in the specific dimensions of social isolation captured, including living alone, frequency of contact, frequency of social activities, social network size, and family/friendship networks. Additionally, some studies directly compared multiple dimensions of social isolation and reported divergent findings across these measures,^55^, ^60^ suggesting that these various dimensions of social isolation may capture distinct components, rather than interchangeable aspects of a single construct. Moreover, in Lee et al.’s^71^ study comparing loneliness operationalized by the UCLA‐LS and loneliness single‐item question from the CES‐D, differing patterns of association were observed between the measures. This indicates that various loneliness measures may not be universally equivalent, and that scale selection may meaningfully impact the detection of neurobiological correlates. It is also important to note that the CES‐D is a depression measure that contains a single item pertaining to feelings of loneliness, which may not be as sensitive in capturing the multidimensional experience of loneliness compared to dedicated loneliness tools such as the UCLA‐LS.

A third source of heterogeneity across the literature pertains to statistical reporting. The lack of consistent effect size reporting and reliance on significance‐based metrics limited comparison of association strength across studies and prevented quantitative synthesis. To be able to conduct meta‐analyses on this subject, authors would need to consistently report standardized effect sies (e.g., correlation coefficients, β coefficients, hazards or odds ratios), corresponding measures of precision (e.g., 95% confidence intervals, standard error), exact *P* values, and clear reporting of analytic models, covariate inclusion, and handling of missing data.

Beyond heterogeneity, an additional methodological consideration concerns the measurement of social isolation. Unlike loneliness and the UCLA‐LS, a gold standard measure for quantifying social isolation does not exist. Currently a variety of metrics and tools have been used to operationalize social isolation (see Table 4), ranging from single‐item questions (e.g., “*How frequently do you socially interact with others?*”; “*How many people do you live with?*”) to established questionnaires. However, although incorporating structural elements of social isolation, tools such as the LSNS^127^ were designed to measure the perceived quality and availability of social ties within social networks. In contrast, the SNI^128^ represents a more objective operationalization of social integration, although it includes a single question pertaining to perceived closeness. Furthermore, as social isolation research continues to gain attention, there is an increasing need for the development of a multi‐dimensional, social isolation–specific measure to ensure accurate assessment and representation of the social construct.

A major challenge consistently faced across the broader field of social health research is the accurate representation of social isolation and loneliness. Historically, these social health constructs were often treated as synonymous but are now recognized as distinct experiences^5^, ^8^. Within the previous few decades, papers have frequently used the terms social isolation and loneliness synonymously, applied measures designed for one concept when investigating the other, used inconsistent definitions, and conflated findings, likely contributing to imprecise and misleading representations of these social concepts (e.g., Zovetti et al.,^25^ Harrington et al., ^29^ and Ishikawa et al.^129^). This issue remains evident, as three recent studies were excluded from this review due to the use of measures conflating social isolation and loneliness,^30^, ^130^, ^131^ emphasizing the importance of clear conceptual distinction and accurate operationalization to minimize this confusion.^5^ These methodological inconsistencies have possibly contributed to the mixed, conflicting, and inconclusive findings observed across the literature (e.g., Lara et al.^132^ Wang et al.,^133^ and Joyce et al.^134^), as inaccurate definitions and measures not only impedes the quality of results but obscures the distinct associations of each social concept. In the context of dementia, continued conflation further hinders our ability to identify and differentiate the unique neurobiological mechanisms and pathways through which social isolation and loneliness may contribute to dementia risk.

Furthermore, several key recommendations emerge for future research in this field. As seen in Table 3, relatively fewer studies directly compared the neurobiological associations of both social isolation and loneliness within a single study design. Hence, increasing the use of such approaches would improve the comparability of findings. Future research should also prioritize longitudinal designs and greater inclusion of older adults, to better characterize the temporal nature of neurobiological pathways underlying these associations and their relevance to dementia risk.

### 4.5 Limitations

This systematic review has a few limitations. Despite rigorous efforts, it is possible that some relevant papers may have been missed. As non‐English articles were excluded, the generalizability of these findings may be limited. Additionally, the limited number of studies examining both social isolation and loneliness in the same study design restricted our ability to draw direct comparisons. Consequently, the present review is confined to identifying broader patterns of association rather than inferring definitive neurobiological specificity. Furthermore, the majority of included papers incorporated a cross‐sectional study design, limiting the ability to infer temporal or causal relationships among social isolation, loneliness, and neurobiological outcomes. Finally, as a quantitative synthesis was not feasible due to the heterogeneity in study designs, operationalizations of social isolation and loneliness, neurobiology measures, and statistical reporting, findings were synthesized using a structured narrative approach incorporating vote counting. Hence, the reported patterns reflect descriptive trends within the current literature, rather than evidence of definitive neurobiological specificity.

## 5 CONCLUSION

This systematic review synthesized the existing literature exploring the underlying neurobiological associations of both social isolation and loneliness in middle–older adult populations, highlighting the shared and exclusive neural correlates. The findings suggest social isolation and loneliness exhibit volumetric disturbances in GM and WM structure, sharing regions involved in various social cognitive functions, while social isolation was somewhat consistently associated with MTG volume across the included studies. However, heterogeneity involving the social concept measures, neurobiological outcomes, and respective neuroimaging techniques used, as well as study design, limits causal inference. Future studies are encouraged to use validated multi‐item loneliness measures, incorporate fMRI and broader WM metrics in older adult samples, and prioritize study designs that examine social isolation and loneliness within the same analytical framework to improve comparability. Moreover, the development of a multidimensional, social isolation–specific measure should be prioritized as the social health field advances.

## CONFLICT OF INTEREST STATEMENT

The authors declare no conflicts of interest. Author disclosures are available in the Supporting Information.

## Supporting information




Supporting Information

Supporting Information
