# Supplementary material for: The underlying neurobiology of social isolation and loneliness in middle‐older adults: A systematic review
Source: Alzheimers Dement. 2026 Oct 28;22(10):e71921. doi: 10.1002/alz.71921 (PMC13714697; doi:10.1002/alz.71921)
Supplement: Supplementary file 2 [file ALZ-22-e71921-s002.docx]

**Supplementary Material**

**Full Search Strategy**

**Loneliness Seach Terms**

| “loneliness” OR “lonely” | "neurobiology" OR “neurology” OR "neural correlates" OR "brain structure" OR "brain function" OR "cognitive function" OR "cortical thickness" OR "white matter" OR "grey matter" OR "functional connectivity" OR "resting-state connectivity" OR "brain activation" OR “cortical connectivity” OR “neural network” OR “brain” | "magnetic resonance imaging" OR "MRI" OR "functional MRI" OR "fMRI" OR "diffusion tensor imaging" OR "DTI" OR "positron emission tomography" OR "PET" OR "single-photon emission computed tomography" OR "SPECT" OR "electroencephalography" OR "EEG" OR “ERP” OR “evoked potentials” OR "near-infrared spectroscopy" OR "NIRS" OR "brain imaging" OR "neuroimaging" OR “imaging” OR “scans” OR “QSM” OR “quantitative susceptibility mapping” OR SWI OR “susceptibility weighted imaging” |
| --- | --- | --- |
| “loneliness” OR “lonely” | "magnetic resonance imaging" OR "MRI" OR "functional MRI" OR "fMRI" OR "diffusion tensor imaging" OR "DTI" OR "positron emission tomography" OR "PET" OR "single-photon emission computed tomography" OR "SPECT" OR "electroencephalography" OR "EEG" OR "near-infrared spectroscopy" OR "NIRS" OR "brain imaging" OR "neuroimaging" OR “imaging” OR “scans” | "amyloid" OR "tau" OR "biomarkers" OR "Alzheimer's disease" OR "cerebral infarction" OR "brain lesions" OR “white matter hyperintensities” OR “WM hyperintensities” OR “cerebral microinfarcts” |
| “loneliness” OR “lonely” | ("brain" OR "neural" OR "neurobiology" OR "central nervous system" OR "CNS" OR "cerebral") | ("neuroinflammation" OR "neuro-inflammatory response" OR "microglial activation" OR "pro-inflammatory cytokines" OR "chronic inflammation") |

Social Isolation Search Terms

| "social isolation" OR "socially isolated" OR "social network" OR "live alone" OR "social interaction" | "neurobiology" OR "neural correlates" “neurology” OR OR "brain structure" OR "brain function" OR "cognitive function" OR "cortical thickness" OR "white matter" OR "grey matter" OR "functional connectivity" OR "resting-state connectivity" OR "brain activation" OR “cortical connectivity” OR “neural network” OR “brain” | "magnetic resonance imaging" OR "MRI" OR "functional MRI" OR "fMRI" OR "diffusion tensor imaging" OR "DTI" OR "positron emission tomography" OR "PET" OR "single-photon emission computed tomography" OR "SPECT" OR "electroencephalography" OR "EEG" OR “ERP” OR “evoked potentials” OR "near-infrared spectroscopy" OR "NIRS" OR "brain imaging" OR "neuroimaging" OR “imaging” OR “scans” OR “QSM” OR “quantitative susceptibility mapping” OR SWI OR “susceptibility weighted imaging” |
| --- | --- | --- |
| "social isolation" OR "socially isolated" OR "social network" OR "live alone" OR "social interaction" | "magnetic resonance imaging" OR "MRI" OR "functional MRI" OR "fMRI" OR "diffusion tensor imaging" OR "DTI" OR "positron emission tomography" OR "PET" OR "single-photon emission computed tomography" OR "SPECT" OR "electroencephalography" OR "EEG" OR "near-infrared spectroscopy" OR "NIRS" OR "brain imaging" OR "neuroimaging" OR “imaging” OR “scans” | "amyloid" OR "tau" OR "biomarkers" OR "Alzheimer's disease" OR "cerebral infarction" OR "brain lesions" OR white matter hyperintensities OR WM hyperintensities OR cerebral microinfarcts |
| "social isolation" OR "socially isolated" OR "social network" OR "live alone" OR "social interaction" | ("brain" OR "neural" OR "neurobiology" OR "central nervous system" OR "CNS" OR "cerebral") | ("neuroinflammation" OR "neuro-inflammatory response" OR "microglial activation" OR "pro-inflammatory cytokines" OR "chronic inflammation") |

**Supplementary Table 1**. *Appraisal tool for Cross-Sectional Studies (AXIS) Risk of Bias for Cross-Sectional Studies.*

| **Study** | Introduction | Methods | Results | Discussion/  Conclusion | Other | Risk |
| --- | --- | --- | --- | --- | --- | --- |
| **Cao et al., 2026** | 100% | 80% | 60% | 100% | 100% | Low  80% |
| **D’Agostino et al., 2019** | 100% | 70% | 60% | 100% | 100% | Low  75% |
| **d’Oleire et al., 2018** | 100% | 60% | 60% | 100% | 100% | Low  75% |
| **de Lange et al., 2021** | 100% | 70% | 60% | 100% | 100% | Low  75% |
| **Donovan et al., 2016** | 100% | 70% | 60% | 100% | 100% | Low  75% |
| **Düzel et al., 2019** | 100% | 60% | 60% | 100% | 100% | Low  70% |
| **Flatt et al., 2015** | 100% | 70% | 40% | 100% | 100% | Low  70% |
| **Gao et al., 2020** | 100% | 70% | 60% | 100% | 100% | Low  75% |
| **Gao et al., 2024** | 100% | 80% | 80% | 100% | 100% | Low  85% |
| **Groechel et al., 2024** | 100% | 70% | 60% | 100% | 100% | Low  75% |
| **Hirabayashi et al., 2023** | 100% | 70% | 60% | 100% | 100% | Low  75% |
| **Hou et al., 2021** | 100% | 60% | 60% | 100% | 100% | Low  70% |
| **Kehrer-Dunlap et al., 2022** | 100% | 60% | 60% | 100% | 100% | Low  70% |
| **Koyama et al., 2021** | 100% | 80% | 60% | 100% | 100% | Low  80% |
| **Lan et al., 2015** | 100% | 60% | 60% | 100% | 100% | Low  70% |
| **Lee et al., 2024** | 100% | 80% | 60% | 100% | 100% | Low  80% |
| **Noonan et al., 2023** | 100% | 70% | 60% | 100% | 100% | Low  75% |
| **Park et al., 2023** | 100% | 60% | 60% | 100% | 100% | Low  70% |
| **Qi et al., 2023** | 100% | 70% | 60% | 100% | 100% | Low  75% |
| **Saris et al., 2022** | 100% | 80% | 60% | 100% | 100% | Low  80% |
| **Shao et al., 2020** | 100% | 80% | 60% | 100% | 100% | Low  80% |
| **Shen et al., 2022** | 100% | 80% | 60% | 100% | 100% | Low  80% |
| **Sin et al., 2018** | 100% | 80% | 60% | 100% | 100% | Low  80% |
| **Solé-Padullés et al., 2022** | 100% | 80% | 60% | 100% | 100% | Low  80% |
| **Spreng et al., 2020** | 100% | 60% | 60% | 100% | 100% | Low  70% |
| **van der Velpen et al., 2023** | 100% | 60% | 60% | 100% | 100% | Low  70% |
| **van der Velpen et al., 2024b** | 100% | 80% | 60% | 100% | 100% | Low  80% |
| **Wong et al., 2016** | 100% | 80% | 60% | 100% | 100% | Low  80% |
| **Zajner et al., 2021** | 100% | 80% | 60% | 100% | 100% | Low  80% |
| **Zapater-Fajari et al., 2025** | 100% | 80% | 60% | 100% | 100% | Low  80% |
| **Zhang et al., 2022** | 100% | 80% | 60% | 100% | 100% | Low  80% |
| **Zhao et al., 2024** | 100% | 80% | 60% | 100% | 100% | Low  80% |

**Supplementary Table 2.** *Joanna Briggs Institute (JBI) Risk of Bias for Cohort Studies.*

| **Study** | Q1 | Q2 | Q3 | Q4 | Q5 | Q6 | Q7 | Q8 | Q9 | Q10 | Q11 | (%) yes | Risk rating |
| --- | --- | --- | --- | --- | --- | --- | --- | --- | --- | --- | --- | --- | --- |
| **Lammer et al., 2023** | + | + | + | − | + | + | + | + | − | + | + | 80% | Low |
| **Murayama et al., 2025** | + | + | − | + | + | + | + | + | − | + | + | 80% | Low |
| **Chen et al., 2022** | + | + | + | − | + | + | + | NA | − | − | + | 70% | Low |
| **Salinas et al., 2022** | + | + | − | + | + | + | + | + | − | − | + | 70% | Low |
| **Terracciano et al., 2025** | + | + | − | − | + | + | + | + | − | − | + | 63% | Moderate |
| **van der Velpen et al., 2022** | + | + | − | + | + | + | + | + | − | − | + | 70% | Low |
| **van der Velpen et al., 2024a** | + | + | − | + | + | + | + | + | − | + | + | 80% | Low |
| **Duan et al., 2017** | + | + | − | + | + | + | + | + | − | − | + | 70% | Low |
| **Shen et al., 2025Δ** | + | + | − | + | + | + | + | + | − | − | + | 70% | Low |

**Supplementary Table 3.** *National Institutes of Health Quality Assessment of Controlled Intervention Studies.*

| **Study** | Q1 | Q2 | Q3 | Q4 | Q5 | Q6 | Q7 | Q8 | Q9 | Q10 | Q11 | Q12 | Q13 | Q14 | (%) yes | Risk rating |
| --- | --- | --- | --- | --- | --- | --- | --- | --- | --- | --- | --- | --- | --- | --- | --- | --- |
| **Ehlers et al., 2017** | + | + | − | − | − | + | + | + | + | − | + | + | + | + | 71% | Low |
